# Widespread Genetic Incompatibilities between First-Step Mutations during Parallel Adaptation of *Saccharomyces cerevisiae* to a Common Environment

**DOI:** 10.1371/journal.pbio.1002591

**Published:** 2017-01-23

**Authors:** Jasmine Ono, Aleeza C. Gerstein, Sarah P. Otto

**Affiliations:** Department of Zoology & Biodiversity Research Centre, University of British Columbia, Vancouver, British Columbia, Canada; University of Bath, UNITED KINGDOM

## Abstract

Independently evolving populations may adapt to similar selection pressures via different genetic changes. The interactions between such changes, such as in a hybrid individual, can inform us about what course adaptation may follow and allow us to determine whether gene flow would be facilitated or hampered following secondary contact. We used *Saccharomyces cerevisiae* to measure the genetic interactions between first-step mutations that independently evolved in the same biosynthetic pathway following exposure to the fungicide nystatin. We found that genetic interactions are prevalent and predominantly negative, with the majority of mutations causing lower growth when combined in a double mutant than when alone as a single mutant (sign epistasis). The prevalence of sign epistasis is surprising given the small number of mutations tested and runs counter to expectations for mutations arising in a single biosynthetic pathway in the face of a simple selective pressure. Furthermore, in one third of pairwise interactions, the double mutant grew less well than either single mutant (reciprocal sign epistasis). The observation of reciprocal sign epistasis among these first adaptive mutations arising in the same genetic background indicates that partial postzygotic reproductive isolation could evolve rapidly between populations under similar selective pressures, even with only a single genetic change in each. The nature of the epistatic relationships was sensitive, however, to the level of drug stress in the assay conditions, as many double mutants became fitter than the single mutants at higher concentrations of nystatin. We discuss the implications of these results both for our understanding of epistatic interactions among beneficial mutations in the same biochemical pathway and for speciation.

## Abstract 2

A study of yeast adapted to the fungicide nystatin finds that the first step toward speciation (partial postzygotic reproductive isolation) can evolve rapidly in populations under similar selective pressures.

## Author Summary

We crossed yeast bearing different genetic mutations to determine the fitness of their hybrid offspring. These strains had previously evolved in the presence of the fungicide nystatin. Even though the initial strains had nearly identical genomes, differing only in the mutation they carried within the biosynthetic pathway leading to ergosterol, the hybrid offspring were less fit than expected based on parental fitness. These negative interactions were so strong that beneficial mutations often became deleterious in the presence of one another (sign epistasis). In one third of crosses, the hybrid double mutant grew less well than either single mutant (reciprocal sign epistasis). This work indicates that the first step toward speciation, partial postzygotic reproductive isolation, can evolve rapidly between populations under similar selective pressures, even with only a single genetic change in each.

## Introduction

**Fig 1:**
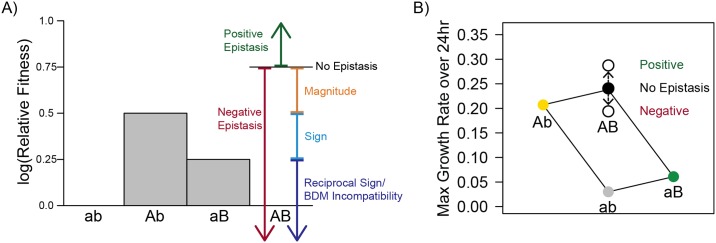
Types of epistatic relationships between mutations. (A) The type of epistasis is observed as the fitness of the single beneficial mutations (A and B) relative to the double mutant (AB). No epistasis occurs when log fitness effects are additive, as shown here (growth rate, our primary fitness measure, is calculated on a log scale). (B) Example plot showing the method used in this paper to illustrate epistatic relationships. The *y*-axis gives maximum growth rate over 24 hours. Point colors indicate strain genotype, where the double mutant is black, the ancestor is grey, and each single mutant has a unique color. Lines are drawn between genotypes that are a single mutational step apart. Without epistasis, the lines form a parallelogram. Epistasis is observed as a double mutant with increased fitness (positive epistasis, higher hollow circle) or decreased fitness (negative epistasis, lower hollow circle).

The number of different evolutionary pathways available to populations adapting to a new environment depends on the range and characteristics of possible genetic solutions. Even populations adapting to the same environmental challenge can diverge genetically from each other if different mutations happen to establish. The long-term impact of this initial divergence depends on the fitness interactions between the available alleles that underlie adaptation to a given environment (“epistasis”). Epistasis can run the gamut from alleles that interact positively and augment each others’ fitness (“positive epistasis") to those that have negative effects on fitness in the presence of each other (“sign epistasis” [1]) (Fig 1).

### Epistasis and Its Role in Evolution

The nature of epistasis is critical to broad-scale evolutionary phenomena. If all possible alleles have the same effect in all genetic backgrounds, we might expect populations that diverge initially to converge to a similar genotype and/or phenotype over time at the fitness optimum. In contrast, if some alleles are beneficial only in certain backgrounds, early genetic changes will limit future genetic options, and populations may diverge genotypically and phenotypically. Thus, the shape and “ruggedness” of the fitness landscape is directly determined by the prevalence of sign epistasis [2–4].

The type of epistasis can also shape the rate of adaptation. In the case of positive epistasis, when early mutations increase the beneficial fitness effects of subsequent mutations, adaptive evolution can accelerate over time. In contrast, when epistasis is negative, i.e., when first-step mutations reduce or oppose the advantage of subsequent mutations, evolution will decelerate. The deceleration of adaptation over time has been previously found in a number of experimental evolution studies [5–8].

Even the formation of new species rests upon epistasis between alleles present in different nascent species. A major driver of postzygotic reproductive isolation between species is the buildup of Bateson–Dobzhansky–Muller (BDM) genetic incompatibilities. These incompatibilities represent reciprocal sign epistasis, where alleles that work well together within a species perform poorly when combined with alleles from the other species in a hybrid individual, leading to hybrid inviability or sterility [9]. Reproductive isolation can also arise from non-reciprocal sign epistasis, where the double mutant is less fit than only one parent, reducing gene flow into the more fit parental population. With enough such asymmetric barriers acting in opposite directions, gene flow may cease entirely between populations.

All models of speciation agree that sign epistasis, and particularly reciprocal sign epistasis, is important for speciation, but they differ on why species carry different alleles. Among the models of speciation by natural selection, the classic explanation, proposed by Darwin [10], is that populations diverge into species because they experience different environments and so adapt in ways that often do not work well together. Because of the focus on environmental differences, this explanation has become known as “ecological speciation” [11]. A contrasting hypothesis, known as “mutation-order speciation” [11], focuses on the chance order in which mutations arise and spread in different populations when facing the same selective environment. Even if the mutational steps that have occurred in each population are independently beneficial, combining mutations across populations need not be.

### Determinants of Epistasis

The specifics of the selective environment(s) likely have a major influence on the nature of epistasis between beneficial mutations. In environments where adaptation can occur via the elimination of a single biosynthetic pathway, complete loss-of-function mutations at one step in the pathway may lead mutations in downstream genes to become irrelevant to fitness. Indeed, Bateson [12] originally coined the term “epistasis” in 1909 to describe this type of interaction, in which the action of one gene was blocked by that of another, and this is primarily how molecular geneticists continue to define the word [13]. Considering instead partial loss-of-function mutations, genotypes combining multiple mutations may be more fit than single mutants if flow through the biosynthetic pathway is reduced by each additional mutation. In either case, we would expect double mutants to have equal or greater fitness than single mutants if knocking out a pathway is beneficial (as long as there are no pleiotropic effects beyond the pathway), and consequently sign epistasis and reproductive isolation should not arise.

On the other hand, if an intermediate phenotype is optimal in a particular environment, mutations that are beneficial on their own may overshoot the optimum when combined, causing a reduction in fitness. In this type of environment, theoretical work predicts that sign epistasis should be particularly frequent between independently selected mutations that have relatively large effects on the phenotype [14].

There is also increasing evidence that epistasis is more often negative for mutations in functionally related genes. In a large-scale screen for genetic interactions in which mutations in most of the ~6,000 genes in the yeast *S*. *cerevisiae* were tested pairwise in 23 million double mutants (including mutations in both nonessential and essential genes), Costanzo et al. [15] found that combinations of genes involved in the same biological process were enriched for negative interactions. This enrichment suggests, counter to intuition, that strongly negative fitness interactions, of the form that give rise to reproductive incompatibilities, may be more likely to accumulate between populations experiencing the same selective environment compared to those experiencing different environments.

### Reproductive Incompatibilities in Nature and in the Lab

To date, few incompatibilities between or within species have been genetically characterized, although recent advances in genomic sequencing technology have greatly aided the discovery of the genetic basis of speciation. For natural populations, the majority of incompatible alleles (“speciation genes”) that have been characterized are found between species adapted to different local environments, presumably representing cases of ecological selection (documented in [16]; see their Tables S1 and S2). For example, the buildup of a suite of plant-specific traits has allowed one species of *Drosophila* to utilize a different, normally toxic, host plant [17], and selection on soils of different salinity has caused the accumulation of quantitative trait loci associated with salt tolerance in a hybrid species of *Helianthus* sunflowers beyond what is found in its parental species [18]. In other cases, genetic incompatibilities between natural populations have been identified for which there is no clear connection to the external selective environment, including BDMs caused by the reciprocal silencing of alternative duplicate gene copies [19] or the differential accumulation of selfish genes and suppressors (see examples in [20]). The exact history of selection is unknown in natural populations, thus it is difficult to know whether these cases represent mutation-order or ecological selection. Natural populations of yeast also show environment-specific genetic incompatibility (including one characterized two-locus BDM [21]), although, as in other taxa, we have no knowledge of the evolutionary history that led to these interactions.

Experimental evolution studies allow direct control over the form of environmental selection, and sign epistasis has been found in some studies that combined mutations from populations adapted to both different and similar selective environments. Dettman et al. [22] evolved different populations of *Neurospora crassa* to high salinity and low temperature. When the evolved strains were mated, lineages adapted to different environments exhibited reduced reproductive success relative to matings between lineages adapted to the same environment, and this reduction was consistent with the action of BDM incompatibilities. A parallel study that examined populations of *S*. *cerevisiae* evolved to high salinity and low glucose for 500 generations found very similar results [23]. Follow-up work identified a BDM incompatibility between an allele of *PMA1* (a proton efflux pump) that arose under high salt adaptation and an allele of *MKT1* (a global regulator of mRNAs encoding mitochondrial proteins) that evolved in low glucose [24]. This was the first reported BDM interaction among known genes isolated from experimentally evolved strains, to our knowledge.

Sign epistasis has also been documented when combining mutations between experimentally evolved populations adapting to the same environment. Kvitek et al. [25] investigated populations of asexually propagated haploid *S*. *cerevisiae* evolved under glucose limitation in continuous culture for 448 generations [26]. Mutations in two genes, *MTH1* and *HXT6/HXT7*, appeared several times in independent lineages during the experiment but never together. These mutations were shown to be individually beneficial, but they had lower competitive fitness when combined in a double mutant than either single mutant or the ancestor, showing reciprocal sign epistasis [25]. Negative epistasis was also prevalent among five additional strains constructed to bear two adaptive mutations that arose in different lineages, with significant negative epistasis in four out of the five comparisons, including one example of sign epistasis [25]. Chou et al. [27] similarly investigated epistasis using an engineered strain of *Methylobacterium extorquens* with a modified central metabolism that was dependent on a foreign pathway artificially introduced on a plasmid. These bacteria were evolved for 900 generations under conditions that utilized this pathway. All adaptive mutations decreased expression of the introduced pathway. Combining mutations, the authors found that expression levels were well predicted by the independent effects of each mutation but that expression mapped nonlinearly onto fitness, leading to sign epistasis in many cases. Collectively, these experiments demonstrate that BDMs can arise rapidly in experimental evolution studies when populations experience either different or similar selective pressures, providing support for both ecological and mutation-order speciation.

### Investigation of Epistasis between First-Step Mutations

What remains unknown from long-term experiments of populations evolved under the same selective pressure is how frequently early adaptive mutations could contribute to reproductive isolation. This raises the question of whether mutation-order speciation occurs because of incompatibilities among mutations that would be beneficial in either population or because the fixation of different initial mutations alters the subsequent selective environment experienced in different populations (i.e., divergent selection due to differences in genetic background).

**Fig 2:**
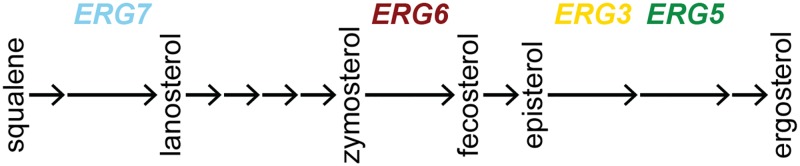
An abbreviated version of the ergosterol biosynthesis pathway. For each gene used in this study, we highlight its position in the ergosterol pathway, with gene names colored according to the scheme used in subsequent figures. Pathway adapted from [29].

We investigate, for the first time, fitness interactions among all pairwise combinations of genes bearing first-step adaptive mutations to a common selective environment. Specifically, we measured epistasis between beneficial mutations acquired in the yeast *S*. *cerevisiae* grown in the presence of the fungicide nystatin [28]. Briefly, Gerstein et al. [28] isolated 35 first-step mutations in 4 μM nystatin, performed genome-wide sequencing, and found that all strains carried a single mutation in one of four genes in the ergosterol biosynthesis pathway (Fig 2; genomic analysis revealed either no or only one other mutation present in the strains used herein, details below). We focused on one mutation in each gene and investigated the fitnesses of all six pairwise double mutants between these four mutations.

For two of these genes (*ERG6* [SGD ID: S000004467] and *ERG3* [SGD ID: S000004046]), many of the mutations found by Gerstein et al. [28] were consistent with a complete loss of function (e.g., early stop codons, similar sterol phenotype to the whole gene knockout). The mutations occurring in the most upstream (*ERG7* [SGD ID: S000001114]) and downstream (*ERG5* [SGD ID: S000004617]) genes in the pathway, however, were not [28]. The *erg7* mutation is a nonsynonymous change close to the end of the gene, and deletion of *ERG7* is inviable. The *erg5* mutation is an in-frame deletion and is unlikely to be a null mutation because the full gene deletion is respiratory deficient [30], which is not observed for this mutant (BMN35 in [28]). Thus, we also assessed whether upstream mutations in the biosynthetic pathway generally mask the effects of downstream mutations or if masking is limited to complete loss-of-function mutations.

Overall, we found that strong negative epistasis, of the type that causes some degree of reproductive isolation between strains fixed for different mutations, was surprisingly common among these first-step mutations. Indeed, the interactions were so negative that they reversed the direction of effect in over half of the double mutants, causing beneficial mutations to become deleterious when in combination and double mutants to be less fit than at least one of the two single mutants (sign epistasis; Fig 1). Furthermore, in one third of the comparisons, the double mutants were less fit than both single mutants (reciprocal sign epistasis). We assayed mutational effects in both haploid and diploid backgrounds, finding similar results regardless of ploidy, indicating that these epistatic relationships are likely to hold across stages of the yeast life cycle. Epistatic relationships for fitness were not well predicted by sterol profiles or pathway position of the mutants, however, suggesting that selection does not simply act via flux through the pathway to ergosterol.

Finally, we investigated epistasis in different concentrations of nystatin to determine how epistatic relationships, and therefore reproductive isolation, might change under different levels of environmental stress. Previous work with antibiotic resistance in bacteria has shown that the shape of fitness landscapes can be strongly dependent on antibiotic concentrations [31]. Interestingly, we found that the negative interactions observed between beneficial mutations at lower concentrations of nystatin reversed sign and became increasingly positive at higher concentrations of nystatin. Indeed, only the double mutants exhibited substantial growth in the higher concentrations of nystatin tested. Thus, although combining single-step mutations generally reduced fitness in the historical nystatin environment, these same combinations were more likely than the individual mutations to allow colonization of even harsher environments.

## Results

### Epistasis of Haploids in Nystatin

We characterized the epistatic interactions between pairs of mutations that act in the ergosterol biosynthesis pathway and individually confer increased fitness when exposed to the antifungal drug nystatin. Maximum growth rate of ancestral, single mutant, and double mutant genotypes was characterized in haploid strains of both mating types in a rich medium composed of yeast extract, peptone, and dextrose (YPD) + 2 μM nystatin (“nystatin2”). Outlier data points were detected statistically and removed from further analyses, although we note where inclusion of outliers would have affected the results (for further details, see the section “Outlier Detection and Removal”). The effect of mating type (and its associated auxotrophy) was not significant (*p* = 0.19), and the data for the two haploid mating types will be considered together, except where noted (see Dryad file for additional statistical methods and results [32]).

**Fig 3:**
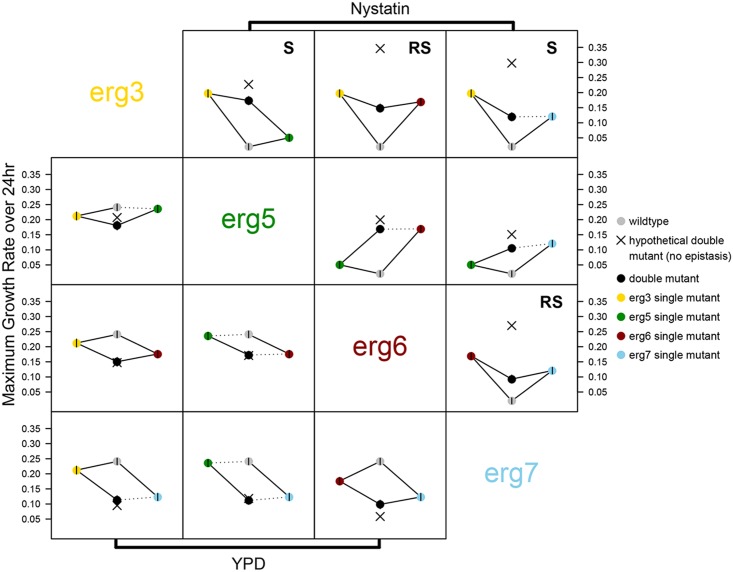
Maximum growth rate of haploid strains in nystatin2 (above diagonal) and YPD (below diagonal). Points are the fitted least-squares means of the maximum growth rates, determined in the mixed-effects model. ×’s denote the additive fitness null expectation for the double mutant, i.e., with no epistasis. Each single mutant is colored differently, the double mutant is black, and the ancestor is grey. Vertical bars represent 95% confidence intervals of the fitted least-squares means. Solid lines indicate significant contrasts between the fitted means, whereas dotted lines are nonsignificant. Combinations showing significant sign (S) and reciprocal sign (RS) epistasis are indicated by the presence of the abbreviation at the top of the panel. In nystatin2, the comparison between *erg3 erg5* and *erg3* is not significant when outliers are included, and the *erg3 erg6* versus *erg6* comparison is only marginally significant (*p* = 0.083). In YPD, comparisons *erg3 erg6* versus *erg6* and *erg6 erg7* versus *erg7* are not significant when outliers are included. All underlying raw data and analyses can be found in Dryad [32].

Using a mixed-effects model, all main effects of individual mutations were positive, confirming that the mutations improved growth in nystatin (Table 1). To assess epistasis, least-squares means of maximum growth rates were inferred from the model and compared between double and single mutants and between single mutants and ancestral strains, correcting for multiple comparisons. Double mutants were never significantly more fit than the best of the single mutants (top right panels in Fig 3), and all pairwise interactions exhibited significant negative (antagonistic) epistasis (Table 1). The double mutant was significantly less fit than the fittest single mutant in four cases (“sign epistasis”: *erg3 erg5*, *erg3 erg6*, *erg3 erg7*, and *erg6 erg7*) and significantly less fit than both single mutants in two cases (“reciprocal sign epistasis”: *erg3 erg6* and *erg6 erg7*, Table 1, Fig 3). The results are similar when fitness is measured by optical density (OD) after 24 hours of growth instead of maximum growth rate over 24 hours (S1 Fig). The strong negative interactions indicate that these alleles, when combined, confer genetic incompatibilities between the strains.

**Table 1:** Results from a mixed-effects model run on all genes using the haploid maximum growth rate data in nystatin2. Coefficients of main effects are the differences in mean maximum growth rate between the single mutant strains and the ancestral strain (difference between *MATα* and *MATa* in the case of mating type). Coefficients of interaction terms are the differences in mean maximum growth rate between the double mutant strains and the sum of the two single mutant coefficients added to the ancestral value. Probabilities (*p*) are the result of an ANOVA between the full model and one lacking that term. The last three columns refer to the type of epistasis present (Fig 1). “Epistasis” indicates a significant departure from an additive model of growth rates, which can be either negative or positive. “Sign” and “Reciprocal sign” refer to cases where the double mutant grows significantly less well than one or both single mutants, respectively.

Term	Coefficient	SE	*p*	Epistasis	Sign	Reciprocal sign
mating type	−0.0034	0.0026	0.19			
erg3	0.18	0.0057				
erg5	0.030	0.0049				
erg6	0.15	0.0049				
erg7	0.10	0.0049				
erg3*erg5	−0.054	0.0090	**3.1 × 10**^**−9**^	negative	✓^a^	
erg3*erg6	−0.20	0.0090	**< 10**^**−15**^	negative	✓	✓^a^
erg3*erg7	−0.18	0.0090	**< 10**^**−15**^	negative	✓	
erg5*erg6	−0.031	0.0076	**4.6 × 10**^**−5**^	negative		
erg5*erg7	−0.046	0.0078	**5.1 × 10**^**−9**^	negative		
erg6*erg7	−0.18	0.0083	**< 10**^**−15**^	negative	✓	✓

Significant *p*-values are in bold.

^*a*^ Not significant when outliers are included.

SE, standard error.

### Comparison of Epistasis between Haploids and Diploids

**Fig 4:**
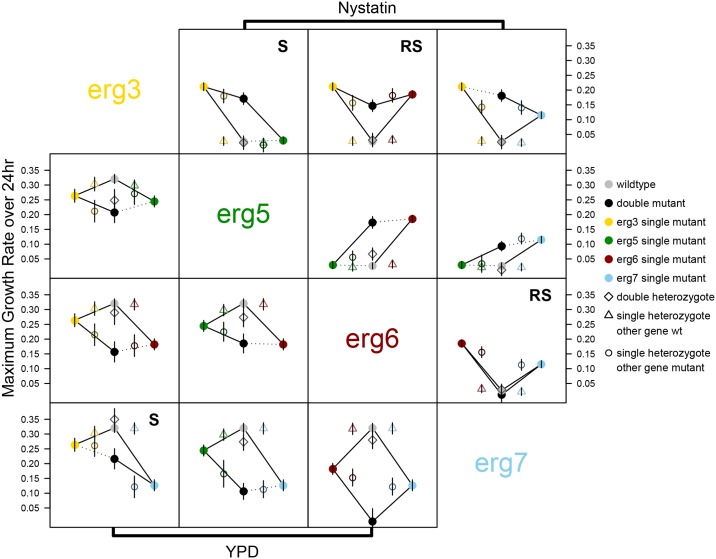
Maximum growth rate of diploid strains in nystatin2 (above diagonal) and YPD (below diagonal). Points are the fitted least-squares means of the maximum growth rates, with closed circles determined in the mixed-effects model including only homozygous strains and open symbols from the model that includes heterozygous strains (open diamonds: double heterozygotes; open triangles: single heterozygotes that are wild type at the other gene; open circles: single heterozygotes that are homozygous mutants at the other gene). Points and bars are otherwise as in Fig 3. All symbols are colored intermediately according to genotype and arrayed along the *x*-axis so as to lie between the two strains that are genotypically most similar to it. Solid lines indicate significant comparisons in tests run including only homozygous strains, whereas dotted lines are nonsignificant comparisons. See S4 Fig for statistical comparisons including heterozygous strains and Fig 3 for further graphical details. In YPD, the homozygous comparison *erg3 erg5* versus *erg3* is not significant when outliers are included. Note that the point for *erg5/ERG5 erg6/erg6* was removed because it was later found to have lost heterozygosity at *ERG5*. All underlying raw data and analyses can be found in Dryad [32].

We characterized epistatic interactions of maximum growth rate for the homozygous diploid strains in nystatin2 and compared them to the haploid results to determine whether the interactions were ploidy dependent. As in haploids, single mutations generally improved the growth of diploid homozygotes in nystatin2, although the *erg5* mutation did not do so significantly in a pairwise comparison with the ancestral strain (Fig 4). Qualitatively, epistatic interactions were also similar to the haploids (Table 2, Fig 4) whether fitness was measured by maximum growth rate or OD after 24 hours of growth (S2 Fig).

**Table 2:** Results from a mixed-effects model run on all genes using the homozygous diploid maximum growth rate data in nystatin2. For statistical and column details, see Table 1.

Term	Coefficient	SE	*p*	Epistasis	Sign	Reciprocal sign
erg3	0.18	0.0065				
erg5	0.0028	0.0058				
erg6	0.16	0.0060				
erg7	0.088	0.0057				
erg3*erg5	−0.043	0.012	**0.00037**	negative	✓	
erg3*erg6	−0.22	0.012	**< 10**^**−15**^	negative	✓	✓
erg3*erg7	−0.12	0.012	**< 10**^**−15**^	negative		
erg5*erg6	−0.015	0.012	0.19			
erg5*erg7	−0.025	0.010	**0.015**	negative		
erg6*erg7	−0.26	0.014	**< 10**^**−15**^	negative	✓	✓

Significant *p*-values are in bold.

SE, standard error.

When we categorized the type of epistasis statistically for maximum growth rate, most interactions were of the same type (sign epistasis: *erg3 erg5*; reciprocal sign epistasis: *erg3 erg6* and *erg6 erg7*; negative epistasis: *erg5 erg7*). There were, however, several quantitative differences. The *erg6 erg7* double mutant was so unfit in diploids that we were often not able to standardize it properly in the growth assays (low growth, as measured by OD, was observed in all concentrations of nystatin tested, S3 Fig). Furthermore, in two cases, epistasis was qualitatively similar, but the differences were no longer statistically significant (sign epistasis: *erg3 erg7*; negative epistasis: *erg5 erg6*).

To visualize the full diploid fitness landscape, we repeated the analysis including all heterozygous strains (open symbols in Fig 4, pairwise comparisons in S4 Fig). Low F_1_ hybrid fitness was typical; double heterozygous strains (open diamonds) were uniformly low in fitness when compared to the homozygous single mutants (not significantly so when compared with the weak *erg5/erg5* mutant). Mutations were generally partially to fully recessive and did not have a large effect on fitness when comparing heterozygotes to wild type at a gene, either when the other gene was wild type (open triangles) or homozygous mutant (open circles).

### Epistasis for Growth in YPD

To determine the extent to which epistasis reflected gross fitness defects not specific to nystatin resistance, we repeated the analysis on maximum growth rate in YPD, a rich growth medium. As in nystatin2, mating type (and its associated auxotrophy) had no significant effect (*p* = 0.98), and results were averaged over mating types.

The single mutations were generally deleterious in YPD (note the negative coefficients for the individual mutations, Tables 3 and 4), consistent with previous characterization of these mutations [28]. The exception is the haploid *erg5* mutant, which is not significantly less fit than the ancestor in a pairwise comparison of maximum growth rates (bottom left panels in Figs 3 and 4). As observed in nystatin2, the double mutant often had lower fitness than the single mutants in YPD, although the strength of epistasis was generally weak (most interactions resemble a parallelogram, Figs 3 and 4). Significant sign epistasis was only observed in a single diploid case (*erg3 erg7*).

**Table 3:** Results from a mixed-effects model run on all genes using the haploid maximum growth rate data in YPD. For statistical and column details, see Table 1. There were no cases of sign epistasis.

Term	Coefficient	SE	*p*	Epistasis
mating type	0.000042	0.0027	0.98	
erg3	−0.029	0.0057		
erg5	−0.0051	0.0049		
erg6	−0.065	0.0049		
erg7	−0.12	0.0050		
erg3*erg5	−0.026	0.0091	**0.0034**	negative
erg3*erg6	0.0030	0.0090	0.74	
erg3*erg7	0.018	0.0091	**0.041**^a^	positive
erg5*erg6	0.0018	0.0077	0.81	
erg5*erg7	−0.0065^b^	0.0079	0.41	
erg6*erg7	0.040	0.0084	**1.77 × 10**^**−6**^	positive

Significant *p*-values are in bold.

^*a*^ Not significant when outliers are included.

^*b*^ Positive when outliers are included.

SE, standard error.

**Table 4:** Results from a mixed-effects model run on all genes using the homozygous diploid maximum growth rate data in YPD. For statistical and column details, see Table 1. There were no cases of reciprocal sign epistasis.

Term	Coefficient	SE	*p*	Epistasis	Sign
erg3	−0.057	0.011			
erg5	−0.076	0.010			
erg6	−0.14	0.010			
erg7	−0.19	0.010			
erg3*erg5	0.020	0.022	0.36		
erg3*erg6	0.032	0.022	0.14		
erg3*erg7	0.15	0.022	**8.5 × 10**^**−11**^	positive	✓
erg5*erg6	0.079	0.020	**5.6 × 10**^**−5**^	positive	
erg5*erg7	0.056	0.018	**0.0021**	positive	
erg6*erg7	0.016	0.025	0.53		

Significant *p*-values are in bold.

SE, standard error.

Epistatic interactions in YPD were qualitatively different from those observed in nystatin2 and often differed between haploids and diploids (Tables 3 and 4). In contrast to the prevalence of negative epistasis in nystatin2, significant positive epistasis was observed in some cases (the double mutant was more fit than expected under the additive model). The low growth in YPD of most double mutant strains suggests that the negative relationships observed in nystatin2 may, in part, be due to intrinsic growth problems, perhaps due to the instability of the cell membrane without proper ergosterol synthesis.

### Tolerance across a Range of Nystatin

To assess whether the genetic interactions depended on the concentration of drug, growth was measured as OD after 24 hours over a range of nystatin concentrations (0, 1, 2, 4, 8, 16, 32, 64, 128, 256 μM). We focused here on OD to assess the range of environments in which the yeast strain could grow, even if slowly, and because of the massive replication required. Although OD is thought to reflect the efficiency of cells’ ability to turn nutrients into cellular material rather than the rate of growth, OD and maximum growth rate were correlated for the single mutants analyzed here [28], and the interactions observed were qualitatively similar for the concentrations of nystatin used in both the maximum growth rate and OD assays (0 μM and 2 μM).

As before, mating type was not found to have a significant effect on OD in the haploid data (linear model that included mating type, concentration of nystatin, and strain identity as fixed effects; mating type: F = 0.23, df = 1, *p* = 0.63; concentration of nystatin: F = 600.12, df = 1, *p* < 10^−15^; strain: F = 31.95, df = 10, *p* < 10^−15^), and data were pooled across mating types.

**Fig 5:**
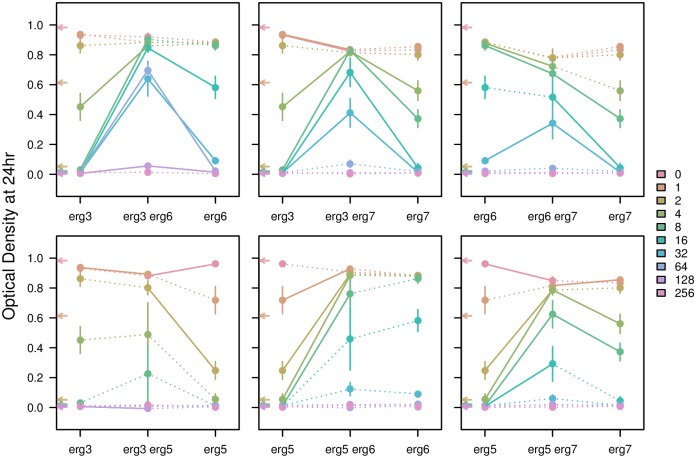
OD after 24 hours of growth for haploid strains in a range of concentrations of nystatin. Colors go from red to purple, through blues, from lowest to highest concentrations of nystatin (in μM). Lines connect different mutants in the same concentration of nystatin. Lines are solid when the difference in OD is significant in a Welch’s *t*-test and dotted when nonsignificant (not adjusted for multiple comparisons). Arrows on the *y*-axes indicate the OD of the ancestral strain. All replicates were averaged, and error bars denote the standard error. All underlying raw data and analyses can be found in Dryad [32].

We found that the form of gene interactions changed when measured over a range of concentrations of nystatin (haploid results: Fig 5). As observed previously, the double mutant generally had equivalent or lower growth than the two parent mutants at low concentrations of nystatin (0–4 μM), but at high concentrations (32–64 μM), the double mutant strains became the only strains able to grow well. That is, a preponderance of negative epistasis shifted towards a preponderance of positive epistasis as nystatin concentrations rose. This dependence of the sign of epistasis on the concentration of the drug (not only on the presence or absence of the drug) indicates that the outcome of mutation or hybridization will depend heavily on the specifics of the environment in which the yeast is found.

Homozygous diploid strains showed qualitatively similar patterns of growth to the haploid strains, with the exception of the *erg6/erg6 erg7/erg7* double mutant (S3 Fig). When we compared all diploid strains (including heterozygous strains), some interesting patterns emerge (S5 Fig). In many cases, the double heterozygous strain exhibited more growth than either single heterozygous strain (as observed by a “bump” in the middle of the figure), particularly at higher concentrations of nystatin. This may indicate a net beneficial effect of carrying two heterozygous mutations or may reflect an increased potential for loss of heterozygosity (LOH). LOH, in which a locus that is initially heterozygous for a mutant allele becomes homozygous, would be beneficial in our fitness assay because being homozygous for either mutant allele increases growth in nystatin (compare middle point in S5 Fig to those second from either end). This may have occurred during the course of the fitness assay, affecting our final measures of fitness. LOH was previously observed for the single heterozygous mutants over a 72-hour timescale [33], and being heterozygous for two mutations may increase the chance of LOH for at least one of the two. The unexpected increase in fitness in the double heterozygotes may also be indicative of an epistatic interaction providing some benefit to having two heterozygous mutations within the ergosterol pathway compared to full recessivity (i.e., no benefit) with only a single heterozygous mutation [33].

### Ergosterol Phenotypes and Map to Fitness

To determine whether epistasis for fitness was consistent with the sterol phenotypes exhibited by the strains, we extracted and measured the sterol profile of all *MATa* strains. In ancestral samples, we see the characteristic four-peaked curve between 240 and 300 nm that is produced by ergosterol and the late sterol intermediate 24(28)dehydroergosterol [34]. Only the latter sterol shows an absorption band at 230 nm, allowing quantification of ergosterol, but we found the peak between 200 and 230 nm to be very sensitive to the standard used (e.g., newly mixed heptane and ethanol versus heptane layer from extraction performed with no yeast cells and ethanol) and thus limit ourselves to a qualitative description of the results.

**Fig 6:**
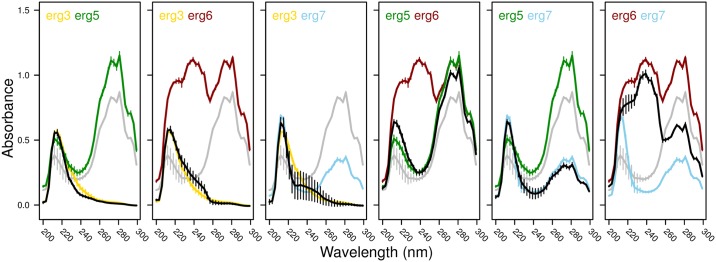
Sterol profiles of all *MATa* haploid strains as measured using a spectrophotometry-based assay. The color scheme is the same as in Fig 3, with the double mutant in black and the ancestral strain in grey. Error bars depict the standard error of three replicates with the exception of *erg6 erg7* (two replicates). The same ancestral and single mutant assays are represented in multiple panels. All underlying raw data and analyses can be found in Dryad [32].

All of our single mutants show similar results to those presented by Gerstein et al. [28] for these same mutants (Fig 6). The two potential loss-of-function mutants (*erg3* and *erg6*) also have similar sterol profiles to knockout mutants of these genes [35, 36]. Double mutants show a variety of profiles, as can be seen in Fig 6. Notably, most double mutants resemble one of the two parent single mutants, with the exception of the *erg6 erg7* double mutant, which is intermediate between the two single mutants in absorbance over much of the measured range (suggesting a mixture of sterols present). All double mutants that include the mutation in *ERG3* tend to show similar profiles to the *erg3* single mutant. Thus, the sterol profiles were not predicted by gene position in the ergosterol biosynthesis pathway (as *ERG6* is upstream of *ERG3*). Furthermore, the similarity in sterol profiles between double and single mutants did not generally predict the patterns observed for maximum growth rate (with the exception of the *erg5 erg7* haploid and diploid, which behaved like *erg7*, and the *erg3 erg7* diploid, which behaved like *erg3*), indicative of a disconnect between sterol profile and fitness.

## Discussion

We investigated the types of genetic interactions present between pairs of first-step beneficial mutations that arose independently in the presence of the fungicide nystatin. We focused on four mutations, representing each gene found to carry a beneficial mutation among 35 strains evolved in 4 μM nystatin [28]. All of these genes are in the biosynthesis pathway leading to the production of ergosterol (the primary sterol in the yeast cell membrane, Fig 2). When ergosterol is bound by nystatin, the cell membrane becomes permeable to ions, sugars, and metabolites [37], and cell death results. When assayed at 2 μM nystatin, the interactions found among these beneficial mutations were predominantly negative, with double mutants exhibiting a lower growth rate in nystatin than expected based on the combined benefits of the single mutations. This negative epistasis was observed in both haploids (Fig 3) and homozygous diploids (Fig 4), supporting previous findings that interactions between mutations in functionally related genes are often negative [15].

### Prevalence of Sign Epistasis

We find that the interactions were so negative that the double mutant grew less well than at least one of the parent single mutants (sign epistasis) in four of the six gene combinations assayed in haploids. In half of these cases, the double mutant grew significantly less well than both single mutants (reciprocal sign epistasis). Similar interactions were observed in diploids (three cases of sign epistasis, two of which were reciprocal). The observation of reciprocal sign epistasis is of particular interest, as this type of BDM incompatibility underlies postzygotic reproductive isolation among speciating lineages. The high frequency of reciprocal sign epistasis observed, even among first-step beneficial mutations acquired in the same environment, confirms the possibility that isolated populations experiencing similar selective pressures can diverge and eventually speciate simply through the order of mutations that happen to arise and fix (mutation-order speciation).

### Maximum Growth Rate in One Environment Does Not Predict Sterol Phenotype or Growth in Other Environments

The prevalence of sign epistasis among our specific set of beneficial mutations is somewhat surprising given the linearity of the biosynthetic pathway in which all of the affected ergosterol genes act (Fig 2). Our results were not consistent with the expectation that the phenotype and fitness of double mutants would be determined by the upstream mutation. In terms of phenotype, the sterol profile of the double mutant was similar to that of the most upstream mutant in only two cases (the *erg5 erg7* and *erg3 erg5* double mutants, Fig 6). In terms of fitness, the growth rate of the double mutant differed significantly from that of the most upstream single mutant in three (haploids) and four (diploids) out of six pairwise comparisons (Figs 3 and 4).

In the combination of two loss-of-function–type mutations (*erg3 erg6*), neither sterol phenotype nor fitness matches that of the upstream (*erg6*) mutation. These results indicate that there remain substantial interactions between the mutations in the ergosterol pathway potentially due to partial activity of the upstream genes creating low levels of substrate for the remainder of the pathway, due to downstream genes acting on alternative sterol substrates, or due to interactions among the intermediate sterols themselves. From previous work in the yeasts *S*. *cerevisiae* and *Candida albicans*, it has been shown that *ERG6* plays a role in offshoot sterol synthesis in mutants of *ERG3* [38], and it is known that intermediate sterols are found at different levels in different compartments of the cell [39] and may impact fitness in a variety of ways (e.g., altering temperature tolerance [40] and virulence [41]).

There was also no clear relationship between sterol phenotype and fitness in these strains. Sterol phenotype for most double mutants resembles one of the two single mutants (Fig 6), but this similarity in sterol phenotype did not generally predict maximum growth rate in nystatin2 (with the possible exception of *erg5 erg7* in haploids and diploids and *erg3 erg7* in diploids, Figs 3 and 4). Future analyses that determine the processing of sterols in the single and double mutants, as well as their pleiotropic effects, would further elucidate these genetic interactions.

Interestingly, the type of epistasis depended strongly on the concentration of nystatin. At lower concentrations of nystatin, similar to those used to acquire the mutations (≤ 4 μM nystatin), epistatic interactions were typically negative (Fig 5), with the double mutant showing similar or lower densities after 24 hours of growth than the single mutants. By contrast, at higher concentrations of nystatin, the interactions were often positive, with double mutants typically able to outgrow both single mutants. Emblematic of this phenomenon, the best growing haploid double mutant strains at 32 μM nystatin (*erg3 erg6*, *erg3 erg7*, *erg6 erg7*) were also those that exhibited the most negative epistasis at lower concentrations. This implies a trade-off between growth at low versus high concentrations of the fungicide. Conceptually, this trade-off suggests that the double mutant initially overshoots the optimum when nystatin concentrations are low, because the costs associated with each ergosterol mutation are combined (perhaps destabilizing the plasma membrane); by contrast, when nystatin concentrations are high, the optimum is shifted even farther away, and extreme reductions in ergosterol and potentially other sterols are needed for the yeast to survive, at which point the double mutant is most fit (see, e.g., Blanquart et al. [42] for a theoretical exploration of this phenomenon). Because membrane damage can trigger cell cycle arrest in yeast [43], another possible explanation for the results observed at high concentrations of nystatin is that single mutants experience cell cycle arrest, reducing growth rate, whereas the additional stress caused by the combination of two mutations and high concentrations of nystatin may cause a checkpoint failure in double mutants, allowing the cells to bypass arrest and continue dividing (personal communication, C. Nislow to J. Ono).

The shifting nature of epistasis as a function of the severity of the environment also has implications for speciation and has not been widely discussed (but see [44] for discussion about environment-dependent epistasis and [45] for an example of an environment-dependent negative epistatic interaction on feeding and growth performance in F_2_ hybrid stickleback). Our results show that BDMs can be environment-specific, and thus gene flow between species might vary according to the environment in which secondary contact occurs [46]. Counterintuitively, our results further suggest that harsher environments may be more conducive to gene flow because of the possible benefit of combining adaptive mutations from different populations. Indeed, environments that are so harsh that only strains combining mutations survive (as we observed at high concentrations of nystatin) might promote hybridization and potentially lead to hybrid speciation (reviewed in [47]). For example, extreme desert environments have selected for combinations of traits that improve drought tolerance, allowing hybrid *Helianthus* sunflowers to colonize and proliferate [48].

### Fitness Landscapes in Haploids and Diploids

Because cell volume to surface area ratios are different for haploids and diploids [49], we might expect differences in growth and epistasis between haploids and diploids, particularly in the face of a selective pressure like nystatin that impacts the cell membrane. By and large, however, our results were consistent across ploidy levels, with diploid homozygous mutants and haploid mutants showing similar patterns of epistasis. One exception was the *erg6/erg6 erg7/erg7* double mutant, which was so unfit in diploids that yields were often too low to obtain initial cell densities similar to other strains in our growth rate assays, even when given multiple extra days of growth. The haploid version of this same double mutant, however, also showed very low fitness. During the initial isolation of the haploids from spores, the double mutant colonies were identifiable by their noticeably smaller size compared to those produced by single mutant and ancestral genotypes. A haploid double mutant strain also exhibited reversion in one instance during growth in 10 mL YPD (Sanger sequencing revealed a secondary mutation in the same codon as the original mutation, reverting the amino acid).

Considering the various diploid heterozygotes, we confirmed that the ergosterol mutations were largely recessive, as found previously for the single heterozygous mutant strains [33]. There were more signs of nystatin resistance in the double mutant strains than in the single mutants, however. One indication of this was the double heterozygous strains showing a slight increase in biomass produced (as measured by OD) compared to the single heterozygous strains across a range of concentrations of nystatin (S5 Fig). Despite this, the double heterozygous strains were uniformly of low growth rate in nystatin2 (open diamonds in Fig 4), with similar sensitivity as found in the ancestor. The generally poor performance of the double heterozygous diploid is of particular interest because this genotype would be the first hybrid product of crosses between strains fixed for different beneficial mutations (see also S4 Fig). Thus, F_1_ hybrid inviability in the double heterozygotes, as well as reciprocal sign epistasis, contributes to reproductive isolation between these strains.

### Implications for Speciation

Overall, we find that the very earliest stages of divergence within a common selective environment can generate postzygotic reproductive isolation, observing sign epistasis, reciprocal sign epistasis, and F_1_ hybrid inviability in double heterozygotes among the first-step adaptive mutations isolated in the presence of nystatin. Although we did not assay incompatibilities at other stages (e.g., meiotic incompatibilities), we expect that further BDMs might be revealed by analyzing other stages in the life cycle (indeed, it was very difficult to sporulate some double mutant strains, particularly *erg5/ERG5 erg7/ERG7*). We speculate that genetic incompatibilities may be especially likely in scenarios such as the one investigated here, in which selection favors large effect mutations. In the initial experiment in which mutations were acquired, the concentration of nystatin was chosen to inhibit growth, so that only mutations capable of rescuing fitness were isolated [28]. Such large effect mutations might have more costly pleiotropic effects and/or be more likely to overshoot the fitness optimum when combined, showing negative epistasis for fitness even if their effects are multiplicative or additive on the underlying trait. If large effect mutations are more likely to interact negatively, which is consistent with our results and others [5–8], short periods of severe selection might be more likely to lead to speciation than longer periods of mild selection. Future experiments comparing genetic incompatibilities among strains with similar levels of divergence but consisting of a few large effect or several small effect genetic differences would be extremely valuable. We also speculate that independent populations experiencing directional selection to the same environmental change might be more likely to speciate than those experiencing directional selection to different environments because the beneficial mutations that accumulate in the former case may be more likely to involve similar pathways and thus be more likely to interact negatively (as has been shown in interaction studies, see [15, 50]). Indeed, even though the beneficial mutations that we assayed were all in the same “linear” pathway and acquired in the same selective environment, we found that the type of epistasis that underlies speciation was common, providing experimental support for the mutation-order speciation hypothesis.

## Materials and Methods

### Strain Construction

We assayed all pairwise interactions in both haploids and diploids between four beneficial mutations acquired in the fungicide nystatin, one in each of *ERG3*, *ERG5*, *ERG6*, and *ERG7* (Table 5). Each mutation was initially isolated in the BY4741 haploid background (*MATa his3*Δ*1 leu2*Δ*0 met15*Δ*0 ura3*Δ*0*, derived from S288C) and struck down to a single colony to remove standing variation. Mutations were detected by whole genome sequencing on an Illumina HighSeq 2000, followed by alignments to the S288C reference strain [28]; few other mutations were detected besides those in the *ERG* genes. For the strains used here, only the strain containing the mutation in *ERG7* also carried a secondary mutation (in *DSC2* [SGD ID: S000005434]), the presence or absence of which did not substantially alter the presented results (see details in S1 File). For a complete description of the isolation of these initial strains, see [28]. All possible haploid and diploid genotypes for each pair of *ERG* genes were created via mating and sporulation. A brief overview of strain construction will be given here, but for a detailed description, see S1 File.

**Table 5:** Beneficial mutations in the strains used for the study of epistasis in the presence of nystatin [28].

Strain	Gene	Genome Position (chr.bp)	Position in Gene (bp)	Mutation	Amino Acid Change
BMN1	*ERG7*	VIII.241194	2097	C→G	Phe699Leu
	*DSC2*	XV.193885	916	G→A	Asp306Asn^a^
BMN9	*ERG6*	XIII.252612	379	G→C	Gly127Arg
BMN32	*ERG3*	XII.254758	898	G→C	Gly300Arg
BMN35	*ERG5*	XIII.302174–302233	253–312	60-bp deletion	

^*a*^ Not known to affect fitness. Encodes a multi-transmembrane subunit of the DSC ubiquitin ligase complex [51, 52]. Null mutant has decreased competitive fitness [53] and decreased resistance to glycolaldehyde [54].

chr.bp, chromosome.base pair; bp, base pair.

To create singly heterozygous strains, each original single mutant strain was mated to BY4739 (*MATα leu2*Δ*0 lys2*Δ*0 ura3*Δ*0*; Open Biosystems), which is isogenic with BY4741 except for the auxotrophies. *MATα* single mutant strains were isolated by sporulation of the heterozygous diploids followed by dissection and testing of the resulting tetrads. Throughout strain construction, histidine and lysine auxotrophies were consistently kept with the same mating types so that all haploid strains were either *MATa his3*Δ*1* or *MATα lys2*Δ*0*. Plates lacking methionine did not efficiently select against the *met15*Δ*0* mutation carried by the original single mutant strains, suggesting a weak effect of this mutation, and the methionine auxotrophy was not tracked.

The *MATα* single mutant strains were then mated to the original *MATa* single mutant strains to create strains that were either homozygous for one mutation or heterozygous for two mutations. The haploid double mutant strains were created through sporulation and dissection of the doubly heterozygous strains. All haploid double mutant strains were confirmed by Sanger sequencing.

We failed to obtain the *MATa erg5 erg6* double mutant haploid strain through crossing and sporulation because the two genes are linked (48 kb away, flanking the centromere of chr XIII). For this strain, a transformation was performed by electroporation using a protocol based on [55] to insert the mutation within *ERG6* into the *MATa erg5* genetic background; this mutation was then checked by Sanger sequencing.

Strains with one heterozygous and one homozygous mutant locus as well as double homozygous mutant strains were created by mating the *MATa* single mutant and double mutant strains to the *MATα* double mutant strains.

A diploid ancestral strain was created by mating BY4741 and BY4739.

### Growth Rate Assays

We conducted a set of growth rate (fitness) assays under nystatin stress and in rich medium (YPD). The experimental design sought to ensure that data were gathered for each combination of wild-type and mutant strains across batches performed on different days. Specifically, within a batch, for a given pair of mutations in haploids and for each mating type, each ancestral strain and each single mutant was assayed twice, while each double mutant was assayed four times (the double mutant was assayed more often because it was the only genotype unique to that pair of mutations). For each pair of mutations in diploids, all possible combinations of the two genes in both heterozygous and homozygous forms (including the nonmutant) were present twice within a batch.

We measured growth in YPD and YPD + 2 μM nystatin (“nystatin2”) using the Bioscreen C Microbiological Workstation (Thermo Labsystems), which measures OD in 100-well honeycomb plates. Nystatin2 was used to assay fitness, because previous studies with these mutants found that 2 μM nystatin inhibits the growth of the ancestral strains while also allowing the growth of all mutant strains [56]. OD was measured automatically using the wideband filter at 30-min intervals for 24 hours from cultures growing at 30°C with maximum continuous shaking. Longer assays were avoided because mutations and LOH events began to accumulate [33]. The maximum growth rate over 24 hours was determined by the spline with the highest slope from a loess fit through natural log transformed OD data, using a custom script written by Richard FitzJohn in R [57] (see Dryad for code [32]).

For complete details on how strains were initially grown from frozen and standardized (“pre-assays”) before measuring growth (“assays”), see S1 File. Briefly, each yeast replicate was grown from frozen in YPD + 0.5 μM nystatin in 100-well honeycomb plates for 72 hours in the pre-assays unless very poor growth of the strain required otherwise, and OD was then determined. YPD + 0.5 μM nystatin was used to help prevent reversion of strains with severe growth defects in YPD and was not found to affect subsequent measures of growth compared to a pre-assay in YPD (the first pre-assay was conducted in YPD, see details in S1 File). For the main assays, honeycomb plate wells were filled with 148.5 μL of YPD or nystatin2. The yeast was then transferred from the pre-assay plates into one well each of YPD and of nystatin2, with the volume transferred determined by the maximum pre-assay OD reading (the minimum volume transferred was 1.5 μL, while the maximum was 7.5 μL). Note that these transfers decreased the concentration of nystatin in the individual wells but never by more than 0.1 μM. Strains were randomized within plates using the same map for the pre-assays and assays in a given batch.

There were not equivalent numbers of replicates for all strains after omitting some data due to low growth (if the volume to be transferred to the assay plate exceeded 7.5 μL), lack of growth, mechanical error, or because some strains had to be re-run (for details, see S1 Table). Nevertheless, at least two replicates per day on at least 2 days were measured for all strains in each medium (with the exception of *erg5/erg5 erg6/erg6*, for which 14 replicates were all run on a single day, S1 Table; for exact numbers and days on which the replicates were run, see Dryad [32]). Although the different numbers of replicates led some crosses to have less power than others, the cross with the least amount of data (*erg6* by *erg7*) was also the one in which the double mutant was particularly unfit, which contributed to the difficulties in assaying fitness but also meant that epistasis was readily detected. In all cases, data for each double mutant was collected simultaneously with data on the ancestor and single mutants, allowing day effects to be factored out in the analysis.

### Tolerance across a Range of Nystatin

Growth at different concentrations of nystatin was assessed following similar procedures to the growth rate assays. To prepare the strains for tolerance assays, pre-assays were again conducted to standardize initial cell concentrations. Stocks were first grown from frozen in four 96-well plates filled with 198 μL of YPD + 0.5 μM nystatin and inoculated with 2 μL of frozen culture. Strains were distributed among the four plates so that there was one replicate of the entire balanced design per plate, randomized within plate. In order to fit all strains on a single plate, some strains were excluded (*MATa erg5 erg6* and *MATa erg3 erg5*). These strains were chosen because initial assays indicated that these double mutants most closely resembled the stronger (non-*erg5*) single mutant. The plates were covered with aluminum lids and incubated at 30°C with continuous shaking at 200 rpm in a container with wet paper towels to minimize evaporative water loss. Prior to removal of the aluminum lid, plates were always spun for 1 min at 3,700 rpm to ensure that all liquid was collected at the bottom.

After 72 hours, all wells were manually mixed and OD was measured on a BioTek plate reader at 630 nm. The well with the minimum OD value among the four pre-assay plates was identified and used to calculate the amount of YPD to add to each pre-assay well to standardize cell density across cultures. Wells containing only medium, those containing *erg6/erg6 erg7/erg7* (see below), and one well that appeared not to have been inoculated were excluded from standardization. Two μL from each well was used to inoculate the assay plates. Assay plates were prepared with 198 μL of YPD + 0, 1, 2, 4, 8, 16, 32, 64, 128, and 256 μM nystatin, with four plates per concentration. The assay plates were covered with aluminum lids and incubated at 30°C in containers with wet paper towels, shaking at 150 rpm.

Exceptions to the pre-assay protocol had to be made for strains with slower growth. Ten ml of 0.5 μM nystatin was inoculated with 15 μL of *erg6/erg6 erg7/erg7* from frozen 2 days before all other strains were inoculated, allowing additional growth time for this unfit strain. On the day that all other strains were inoculated from frozen, the *erg6/erg6 erg7/erg7* culture was concentrated into ~900 μL (although growth was not observable), and 200 μL of this culture was used to replace the medium from the appropriate wells in the pre-assay plates. In addition, *erg6 erg7* (both *MATa* and *MATα*) and *erg6/erg6* were inoculated with 2.67 μL of frozen culture (as opposed to the 2 μL used for all other strains) to compensate for their lower growth rate from frozen.

Twenty-four hours after inoculation, the aluminum lids were removed, wells were manually mixed, and the OD of each assay plate was read on a BioTek plate reader at 630 nm. Some wells had lost volume due to cracks that had developed in the plates, and these wells were omitted from analysis. Prior to analysis, the OD of the medium itself was subtracted from the final OD measurements.

### Sterol Assay

To determine whether the sterol profiles of the single mutants, along with their position within the ergosterol pathway, predict the sterol profiles of the double mutants and whether differences in sterol profiles predict differences in fitness, a spectrophotometry-based assay was used to compare the sterol profiles of the ancestral, mutant, and double mutant *MATa* strains. Sterols were extracted using the alcoholic potassium hydroxide method [34], as previously performed on the single mutant strains [28]. *MATa* strains were struck from frozen onto YPD plates and grown for 65 hours. Three colonies for each strain were inoculated into two separate tubes filled with 10 mL of YPD (total of 20 mL per replicate) and incubated at 30°C on a rotor for 48 hours.

After growth, cells were harvested by centrifugation at 2,700 rpm for 5 min, combining culture from the two tubes by performing two successive spins. The pellets were washed twice with sterile distilled water and 1.2 mL of 25% alcoholic potassium hydroxide was added to each. The tubes were vortexed for 1 min then incubated in an 80°C water bath for 1 hour. After cooling the samples to room temperature, 0.4 mL of sterile distilled water and 1.2 mL of n-heptane were added to each sample, and the tubes were vortexed for 3 min. Samples were collected by taking 220 μL of the heptane layer and adding it to 880 μL of 95% ethanol in a 1.5-mL tube. These tubes were stored at −20°C for 2 days before reading the absorbance every 3 nm between 200 and 300 nm in a quartz microcuvette using a Thermo BioMate 3 spectrophotometer. Due to a posteriori observations that different heptane/ethanol mixtures led to different peak heights near 220 nm, we chose to use one replicate of the *erg6 erg7* strain that showed no evidence of growth (suggesting an inoculation failure), but was otherwise identically treated, as a control for standardization. As a result, only two replicates of *erg6 erg7* are presented.

### Outlier Detection and Removal

Outliers in microbial fitness assays often represent either contamination by a different strain or evolution over the course of the fitness assay. In order to prevent these events from having undue influence on our analyses, we detected outliers for maximum growth rate after omitting some wells due to lack of growth and mechanical error (see details in S1 File). For outlier detection, we first normalized for plate within each day. We did so by finding the global mean maximum growth rate for all ancestral strains over all days and calculating the difference between this and the mean of all ancestral strains on a given plate, yielding a plate correction value. This correction value was added to the maximum growth rate for each strain from the corresponding plate. Outliers were detected by performing a two-sided Grubbs test, allowing us to detect a maximum of one outlier per strain and medium, using the R package *outliers* and the method *grubbs*.*test* [57, 58]. A total of eight replicates in nystatin2 and six replicates in YPD were marked as outliers and removed from all presented statistical and graphical analyses.

All qualitative relationships between strains and the main statistical conclusions were insensitive to the exclusion or inclusion of the identified outliers, with two main exceptions for the haploids in nystatin2 (see S6 and S7 Figs for versions of Figs 3 and 4 that include all outliers). These exceptions are noted in the Results and described in detail in S1 File.

### Statistical Analyses

Epistasis for maximum growth rate was assessed with mixed-effects models run on either all haploid or all diploid strains together, including the genotype at each gene, their pairwise interactions, and mating type (for the haploids) as fixed effects and plate within day as a random effect, fit using restricted maximum likelihood with the *lmer* function from the *lme4* package in R [57, 59]. For diploids, the models were first run using only strains that were homozygous (either mutant or ancestral) for comparison to the haploid data. Significance of interaction terms (and mating type) was determined by performing an ANOVA between the full model and a model dropping that term using the *anova* function in R and fitting models using maximum likelihood.

To determine the type of epistasis present for each pair of genes, the package *lsmeans* [60] was used to both determine the least-squares mean for each strain in the model and to make comparisons between strains using the *contrast* function. The type of epistasis was determined by comparing the double mutant to each single mutant and each single mutant to the ancestor, and only these planned comparisons were performed. The *p*-value was adjusted for the number of tests performed using the multivariate *t* distribution (*mvt* method) in *lsmeans*. To be conservative, we based our categorization of epistasis solely on statistically significant differences. For example, if the double mutant had a lower growth rate than both single mutants but this difference was only significant in one of the two cases, it was considered an example of sign epistasis (significantly lower than one single mutant but not the other) rather than reciprocal sign epistasis.

A similar procedure was then undertaken including heterozygous diploid strains. A model was run using the *lmer* function including all diploid strains together, with plate within day as a random effect. Least-squares means were determined for all diploid genotypes from this model, and comparisons were performed between each diploid genotype and all other diploid genotypes that were one mutational step away. The double heterozygous strains were compared to all other strains for that pair of genes because the potential progeny of the double heterozygote includes all possible genotypes and these comparisons are therefore of biological interest.

For the tolerance assay assessed across a range of concentrations of nystatin, we performed Welch’s *t*-tests of OD after 24 hours between each double mutant and its single mutant parents (day effects were not estimated as all measurements were gathered on the same day). Because we were focused on the changing nature of epistasis, rather than any particular pairwise comparison, a correction for multiple comparisons was not performed.

Data and analyses deposited in the Dryad repository: http://dx.doi.org/10.5061/dryad.vs370 [32].

## Supporting Information

S1 Fig OD after 24 hours of growth for haploid strains in nystatin2 (above diagonal) and YPD (below diagonal), plotted on a log scale. Points are the fitted least-squares means of the ODs, determined in the mixed-effects model run using log(OD). ×’s denote the additive fitness null expectation for the double mutant, i.e., with no epistasis. Each single mutant is colored differently, the double mutant is shown in black, and the ancestor is grey. Vertical bars represent 95% confidence intervals of the fitted least-squares means. Solid lines indicate significant comparisons, whereas dotted lines are nonsignificant comparisons. Combinations showing significant sign (S) and reciprocal sign (RS) epistasis are indicated by the presence of the abbreviation at the top of the panel. The same outliers were removed as in the analysis of maximum growth rate because their growth rates indicate a potential problem with the replicate. Sign epistasis is less often detected in this analysis of log(OD) in nystatin2, likely because even slower growing strains are given time to catch up in cell density over 24 hours. All underlying raw data and analyses can be found in Dryad [32].
(TIF)

S2 Fig OD after 24 hours of growth for diploid strains in nystatin2 (above diagonal) and YPD (below diagonal), plotted on a log scale. Points are the fitted least-squares means of the ODs, with closed circles determined in the mixed-effects model run using log(OD) including only homozygous strains and open symbols from the model that includes heterozygous strains (open diamonds: double heterozygotes; open triangles: single heterozygotes that are wild type at the other gene; open circles: single heterozygotes that are homozygous mutants at the other gene). Points and bars are otherwise as in S1 Fig. All symbols are colored intermediately according to genotype and arrayed along the *x*-axis so as to lie between the two strains that are genotypically most similar to it. Solid lines indicate significant comparisons in tests run including only homozygous strains, whereas dotted lines are nonsignificant comparisons. See S1 Fig for further graphical details. The same outliers were removed as in the analysis of maximum growth rate because their growth rates indicate a potential problem with the replicate. Sign epistasis is less often detected in this analysis of log(OD) in nystatin2, likely because even slower growing strains are given time to catch up in cell density over 24 hours. Note that the strain *erg5/ERG5 erg6/erg6* was later found to be homozygous for the mutation in *ERG5*, likely due to an LOH event. All underlying raw data and analyses can be found in Dryad [32].
(TIF)

S3 Fig OD after 24 hours of growth for homozygous diploids in a range of concentrations of nystatin. These results are qualitatively similar to the haploid strains with the exception of the *erg6/erg6 erg7/erg7* double mutant, which has very low growth in all concentrations of nystatin. Colors go from red to purple, through blues, from lowest to highest concentrations of nystatin. Lines connect different mutants in the same concentration of nystatin. Differences in OD between mutants were not tested statistically and are all represented by solid lines (in contrast to Fig 5). Arrows on the *y*-axes indicate the OD of the ancestral strain. All replicates were averaged, and error bars denote the standard error. Note that tolerance was assayed in the *erg5/erg5 erg6/erg6* homozygous double mutant before we determined that it was likely polymorphic; these points may thus be underestimates (see S1 Table for details). All underlying raw data and analyses can be found in Dryad [32].
(TIF)

S4 Fig Maximum growth rate of diploid strains for each gene combination in nystatin2. Genotype at each of the two genes combined is represented along the *x*- and *y*-axes, with the ancestral genotype in the lower left corner and the homozygous double mutant genotype in the upper right corner. Least-squares means of maximum growth rates, as determined from a model including all possible diploid genotypes, are represented by the darkness of the boxes. Arrows indicate significant differences between genotypes, with arrowheads pointing to the significantly higher growth rate as determined by pairwise comparisons corrected for multiple comparisons using the multivariate *t* distribution in *lsmeans*, as was done for the haploids and homozygous diploids. Only adjacent genotypes on the grid (horizontal and vertical) were compared, with the exception of the double heterozygous strain (center), which was compared to all other genotypes. Note that the strain *erg5/ERG5 erg6/erg6* was later found to be homozygous for the mutation in *ERG5*, likely due to an LOH event. All underlying raw data and analyses can be found in Dryad [32].
(TIF)

S5 Fig OD after 24 hours of growth for diploid strains in a range of concentrations of nystatin. Colors go from red to purple, through blues, from lowest to highest concentrations of nystatin. Lines connect different mutants in the same concentration of nystatin. Mutant strains are ordered one mutational step apart along the *x*-axis, with the homozygous double mutant at both ends. Sections shaded in grey represent mutants carrying at least one homozygous mutation. Differences in OD between mutants were not tested statistically and are all represented by solid lines (in contrast to Fig 5). Arrows on the *y*-axes indicate the OD of the ancestral strain. All replicates were averaged, and error bars denote the standard error. Note that tolerance was assayed in the *erg5/erg5 erg6/erg6* homozygous double mutant before we determined that it was likely polymorphic; these points may thus be underestimates (see S1 Table for details). Also note that the strain *erg5/ERG5 erg6/erg6* was later found to be homozygous for the mutation in *ERG5*, likely due to an LOH event. All underlying raw data and analyses can be found in Dryad [32].
(TIF)

S6 Fig Maximum growth rate of haploid strains in nystatin2 (above diagonal) and YPD (below diagonal) when including outliers. Points are the fitted least-squares means of the maximum growth rates, determined in the mixed-effects model. ×’s denote the additive fitness null expectation for the double mutant, i.e., with no epistasis. Each single mutant is colored differently, the double mutant is black, and the ancestor is grey. Vertical bars represent 95% confidence intervals of the fitted least-squares means. Solid lines indicate significant comparisons, whereas dotted lines are nonsignificant comparisons. Combinations showing significant sign (S) and reciprocal sign (RS) epistasis are indicated by the presence of the abbreviation at the top of the panel. All underlying raw data and analyses can be found in Dryad [32].
(TIF)

S7 Fig Maximum growth rate of diploid strains in nystatin2 (above diagonal) and YPD (below diagonal) when including outliers. Points are the fitted least-squares means of the maximum growth rates, with closed circles determined in the mixed-effects model including only homozygous strains and open symbols from the model that includes heterozygous strains (open diamonds: double heterozygotes; open triangles: single heterozygotes that are wild type at the other gene; open circles: single heterozygotes that are homozygous mutants at the other gene). Points and bars are otherwise as in Fig 3 and S6 Fig. All symbols are colored intermediately according to genotype and arrayed along the *x*-axis so as to lie between the two strains that are genotypically most similar to it. Solid lines indicate significant comparisons in tests run including only homozygous strains, whereas dotted lines are nonsignificant comparisons. See Fig 3 or S6 Fig for further graphical details. Note that the strain *erg5/ERG5 erg6/erg6* was later found to be homozygous for the mutation in *ERG5*, likely due to an LOH event. All underlying raw data and analyses can be found in Dryad [32].
(TIF)

S1 File Supplemental Materials and Methods. (PDF)

S1 Table Experimental design of the growth rate assays. In each epistasis assay, growth rate was measured in a Bioscreen C over a 24-hour period for a pair of ergosterol mutations (first column) using two replicate wells for each genotype (ancestral, single mutant, double mutant for haploids, including those heterozygous or homozygous for diploids), with the exception of double mutant haploids, which were measured in four replicate wells. Checkmarks indicate that all data from this assay were used, whereas bullets indicate that some strains were omitted (see footnotes).
(PDF)

## Abbreviations

BDM: Bateson–Dobzhansky–Muller
LOH: loss of heterozygosity
OD: optical density
YPD: a rich medium composed of yeast extract, peptone, and dextrose
