# Supplementary figures and images for: Widespread Genetic Incompatibilities between First-Step Mutations during Parallel Adaptation of Saccharomyces cerevisiae to a Common Environment
Source: PLoS Biol. 2017 Jan 23;15(1):e1002591. doi: 10.1371/journal.pbio.1002591 (PMC5256870; doi:10.1371/journal.pbio.1002591)

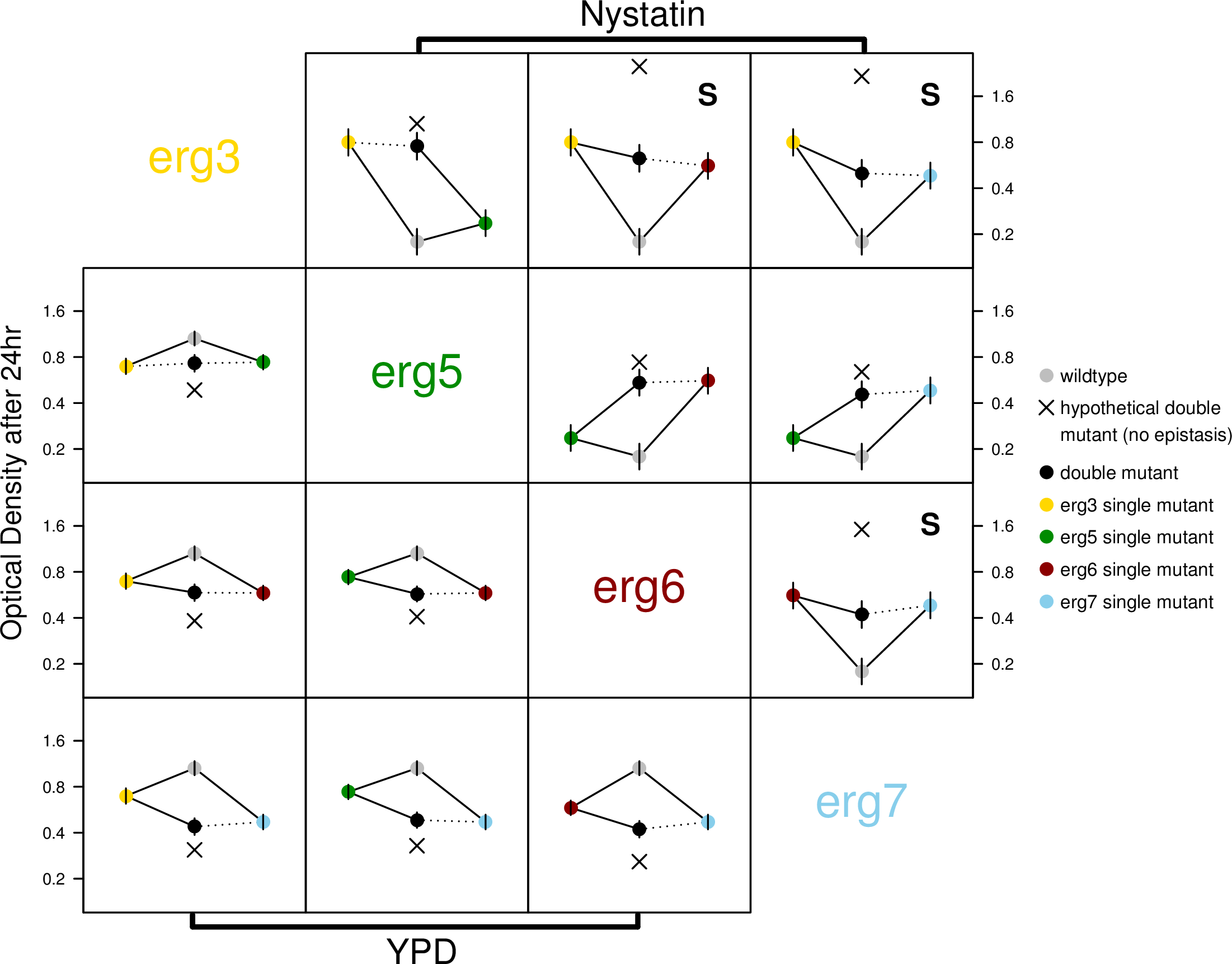

Supplement: S1 Fig — Points are the fitted least-squares means of the ODs, determined in the mixed-effects model run using log(OD). ×’s denote the additive fitness null expectation for the double mutant, i.e., with no epistasis. Each single mutant is colored differently, the double mutant is shown in black, and the ancestor is grey. Vertical bars represent 95% confidence intervals of the fitted least-squares means. Solid lines indicate significant comparisons, whereas dotted lines are nonsignificant comparisons. Combinations showing significant sign (S) and reciprocal sign (RS) epistasis are indicated by the presence of the abbreviation at the top of the panel. The same outliers were removed as in the analysis of maximum growth rate because their growth rates indicate a potential problem with the replicate. Sign epistasis is less often detected in this analysis of log(OD) in nystatin2, likely because even slower growing strains are given time to catch up in cell density over 24 hours. All underlying raw data and analyses can be found in Dryad [32]. (TIF) [file pbio.1002591.s001.tif]

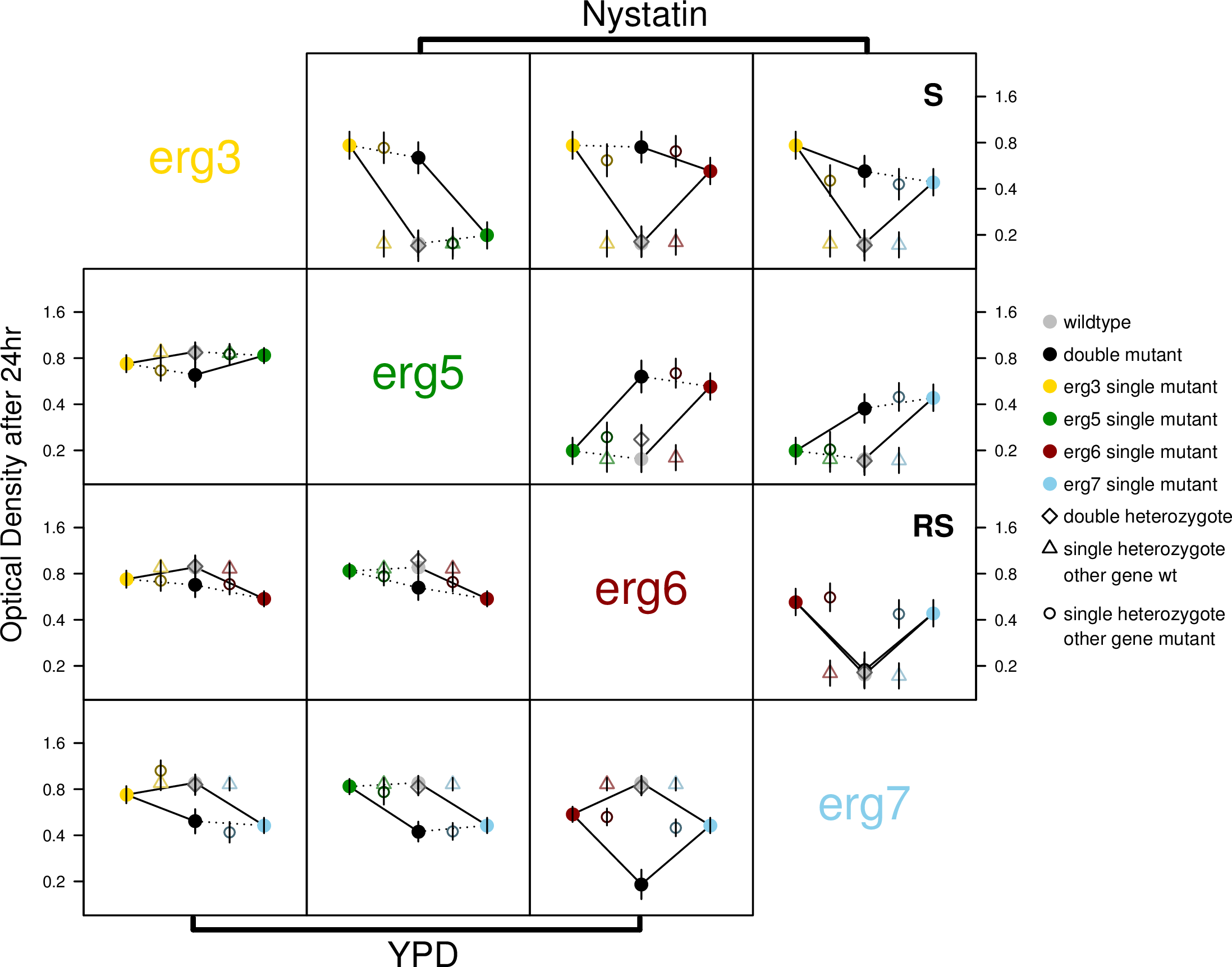

Supplement: S2 Fig — Points are the fitted least-squares means of the ODs, with closed circles determined in the mixed-effects model run using log(OD) including only homozygous strains and open symbols from the model that includes heterozygous strains (open diamonds: double heterozygotes; open triangles: single heterozygotes that are wild type at the other gene; open circles: single heterozygotes that are homozygous mutants at the other gene). Points and bars are otherwise as in S1 Fig. All symbols are colored intermediately according to genotype and arrayed along the x-axis so as to lie between the two strains that are genotypically most similar to it. Solid lines indicate significant comparisons in tests run including only homozygous strains, whereas dotted lines are nonsignificant comparisons. See S1 Fig for further graphical details. The same outliers were removed as in the analysis of maximum growth rate because their growth rates indicate a potential problem with the replicate. Sign epistasis is less often detected in this analysis of log(OD) in nystatin2, likely because even slower growing strains are given time to catch up in cell density over 24 hours. Note that the strain erg5/ERG5 erg6/erg6 was later found to be homozygous for the mutation in ERG5, likely due to an LOH event. All underlying raw data and analyses can be found in Dryad [32]. (TIF) [file pbio.1002591.s002.tif]

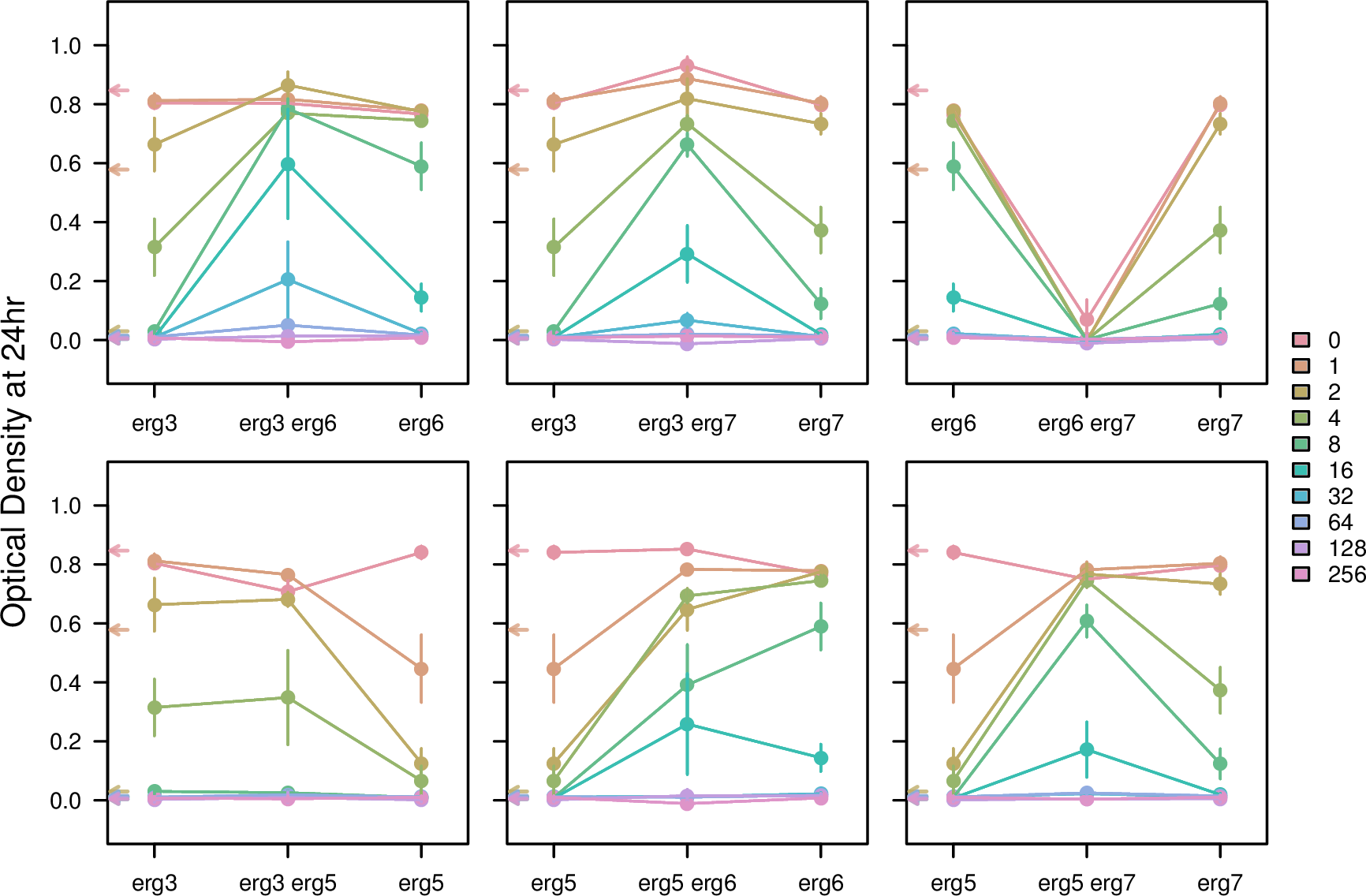

Supplement: S3 Fig — These results are qualitatively similar to the haploid strains with the exception of the erg6/erg6 erg7/erg7 double mutant, which has very low growth in all concentrations of nystatin. Colors go from red to purple, through blues, from lowest to highest concentrations of nystatin. Lines connect different mutants in the same concentration of nystatin. Differences in OD between mutants were not tested statistically and are all represented by solid lines (in contrast to Fig 5). Arrows on the y-axes indicate the OD of the ancestral strain. All replicates were averaged, and error bars denote the standard error. Note that tolerance was assayed in the erg5/erg5 erg6/erg6 homozygous double mutant before we determined that it was likely polymorphic; these points may thus be underestimates (see S1 Table for details). All underlying raw data and analyses can be found in Dryad [32]. (TIF) [file pbio.1002591.s003.tif]

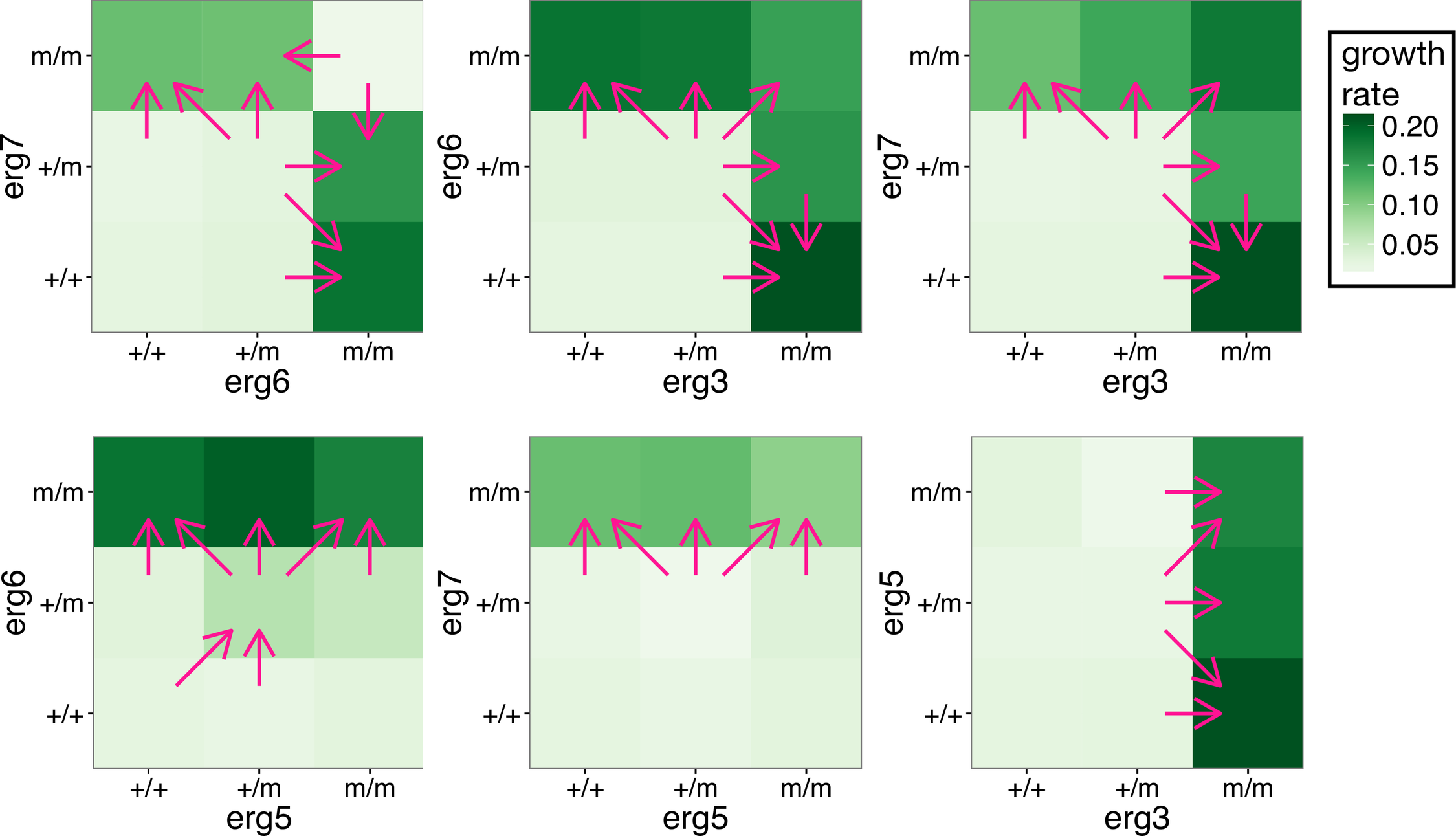

Supplement: S4 Fig — Genotype at each of the two genes combined is represented along the x- and y-axes, with the ancestral genotype in the lower left corner and the homozygous double mutant genotype in the upper right corner. Least-squares means of maximum growth rates, as determined from a model including all possible diploid genotypes, are represented by the darkness of the boxes. Arrows indicate significant differences between genotypes, with arrowheads pointing to the significantly higher growth rate as determined by pairwise comparisons corrected for multiple comparisons using the multivariate t distribution in lsmeans, as was done for the haploids and homozygous diploids. Only adjacent genotypes on the grid (horizontal and vertical) were compared, with the exception of the double heterozygous strain (center), which was compared to all other genotypes. Note that the strain erg5/ERG5 erg6/erg6 was later found to be homozygous for the mutation in ERG5, likely due to an LOH event. All underlying raw data and analyses can be found in Dryad [32]. (TIF) [file pbio.1002591.s004.tif]

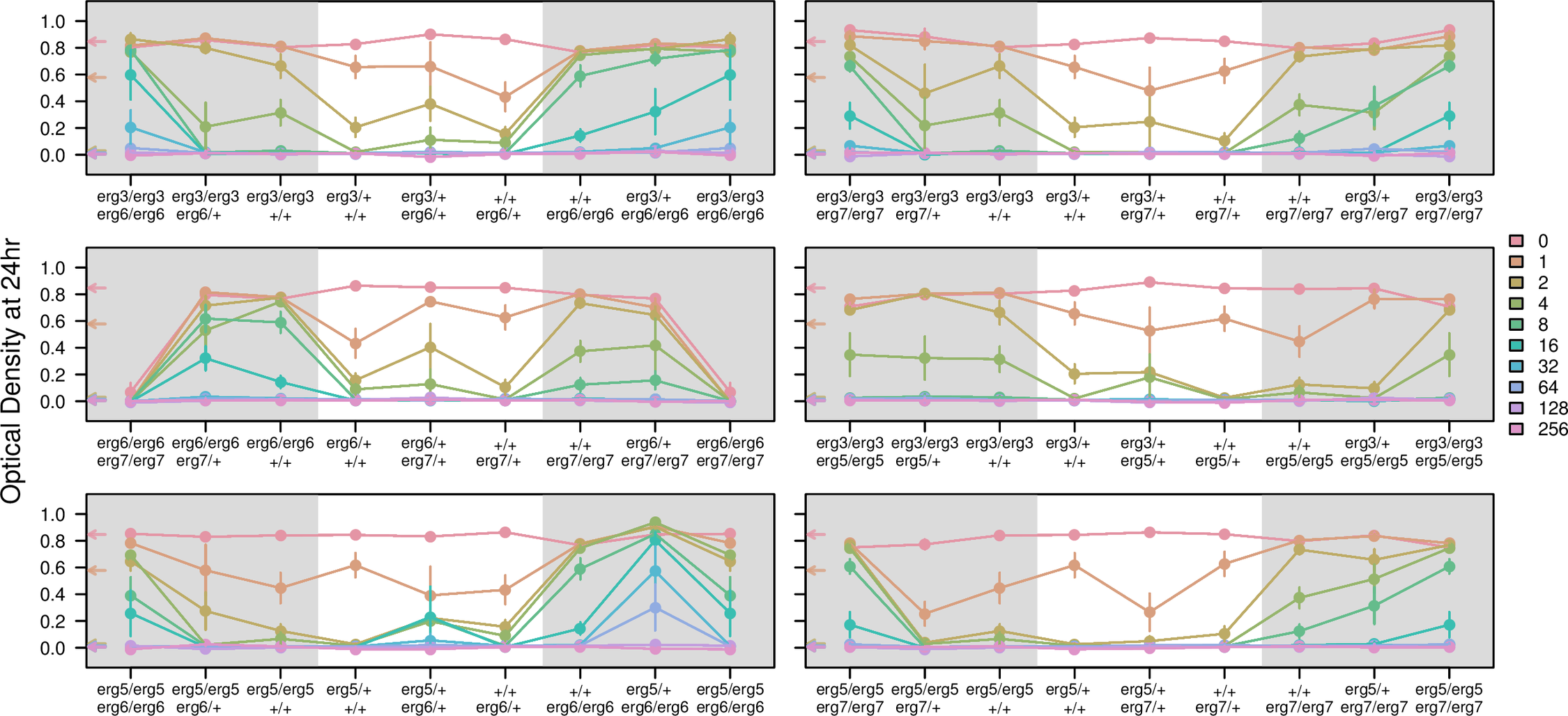

Supplement: S5 Fig — Colors go from red to purple, through blues, from lowest to highest concentrations of nystatin. Lines connect different mutants in the same concentration of nystatin. Mutant strains are ordered one mutational step apart along the x-axis, with the homozygous double mutant at both ends. Sections shaded in grey represent mutants carrying at least one homozygous mutation. Differences in OD between mutants were not tested statistically and are all represented by solid lines (in contrast to Fig 5). Arrows on the y-axes indicate the OD of the ancestral strain. All replicates were averaged, and error bars denote the standard error. Note that tolerance was assayed in the erg5/erg5 erg6/erg6 homozygous double mutant before we determined that it was likely polymorphic; these points may thus be underestimates (see S1 Table for details). Also note that the strain erg5/ERG5 erg6/erg6 was later found to be homozygous for the mutation in ERG5, likely due to an LOH event. All underlying raw data and analyses can be found in Dryad [32]. (TIF) [file pbio.1002591.s005.tif]

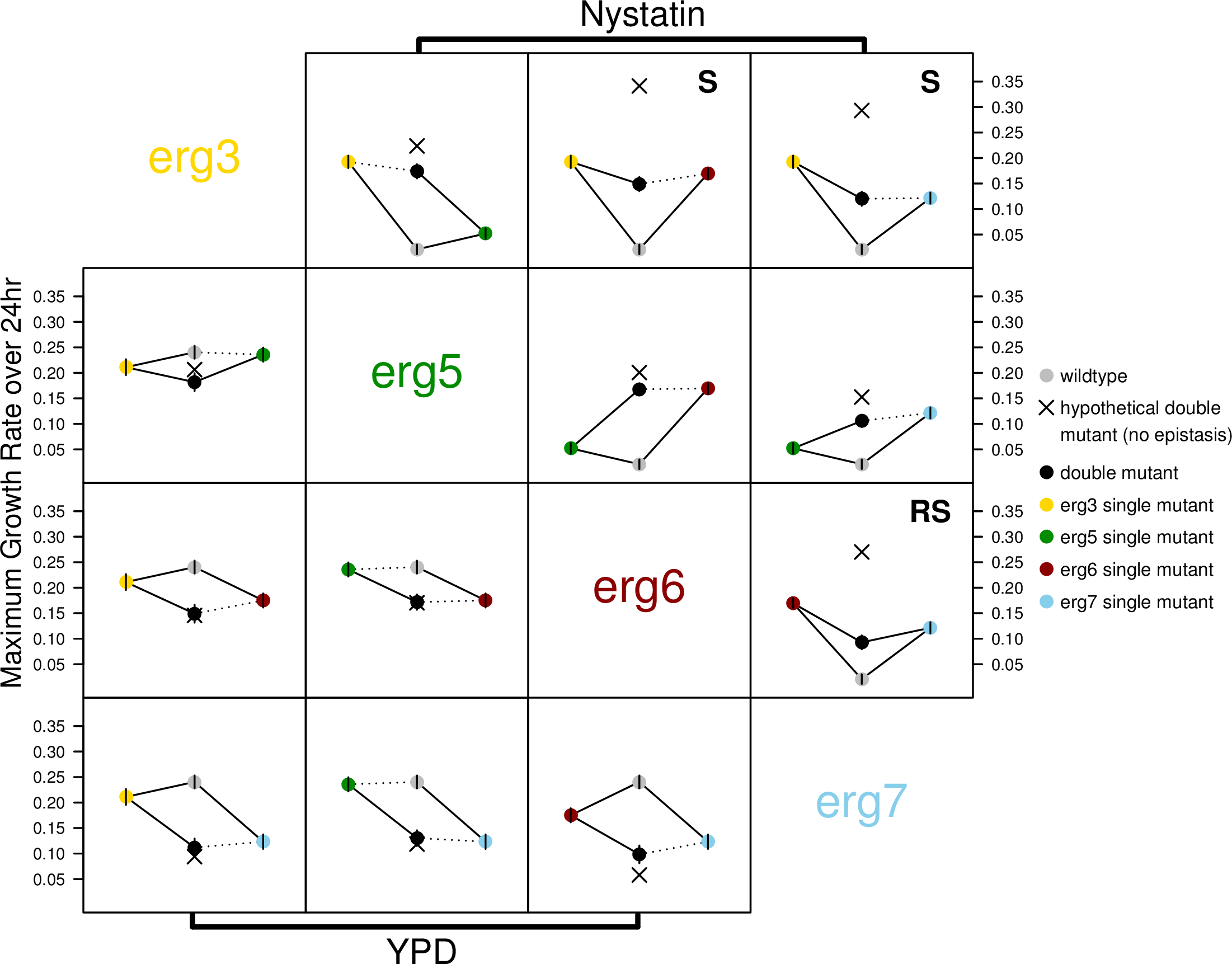

Supplement: S6 Fig — Points are the fitted least-squares means of the maximum growth rates, determined in the mixed-effects model. ×’s denote the additive fitness null expectation for the double mutant, i.e., with no epistasis. Each single mutant is colored differently, the double mutant is black, and the ancestor is grey. Vertical bars represent 95% confidence intervals of the fitted least-squares means. Solid lines indicate significant comparisons, whereas dotted lines are nonsignificant comparisons. Combinations showing significant sign (S) and reciprocal sign (RS) epistasis are indicated by the presence of the abbreviation at the top of the panel. All underlying raw data and analyses can be found in Dryad [32]. (TIF) [file pbio.1002591.s006.tif]

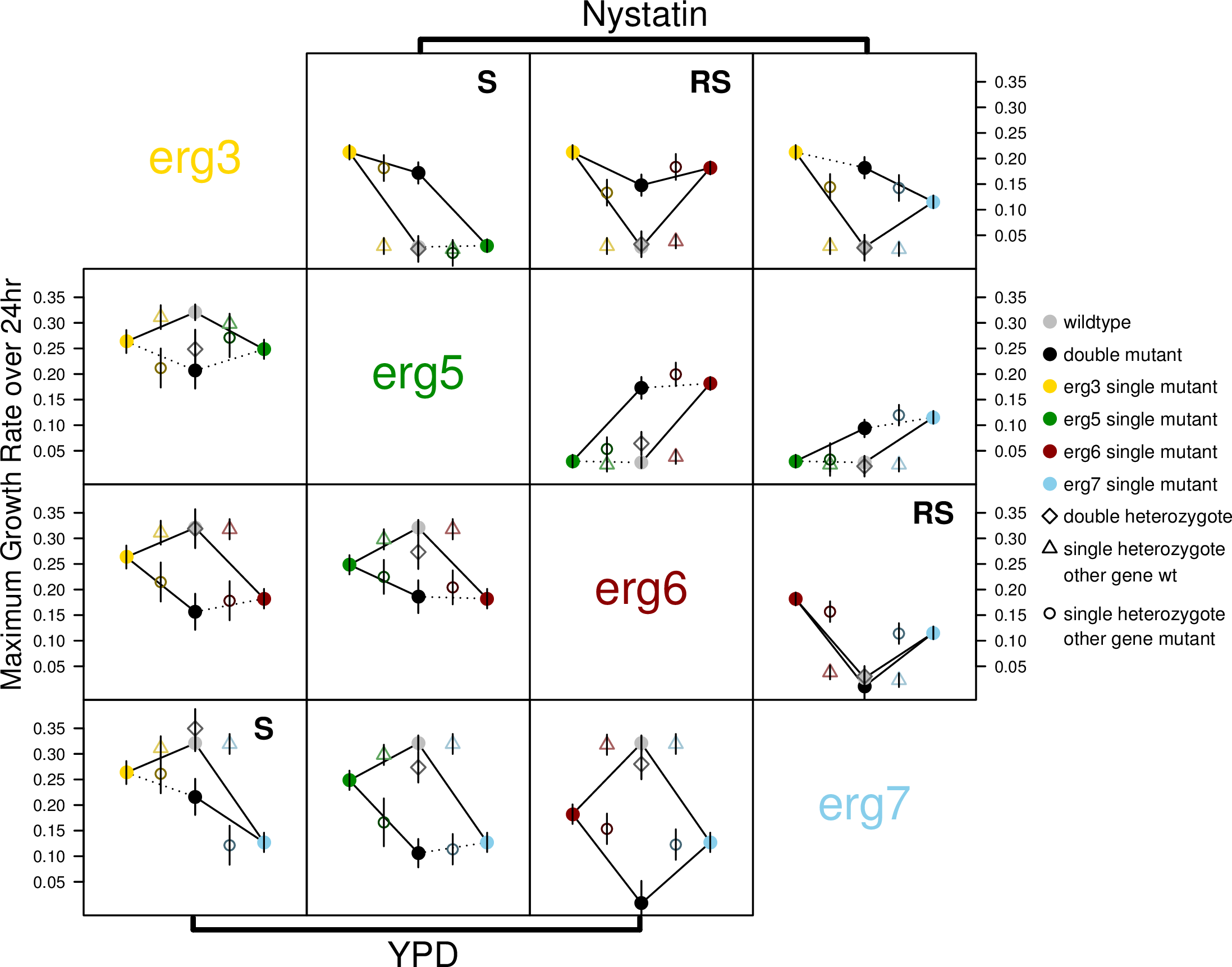

Supplement: S7 Fig — Points are the fitted least-squares means of the maximum growth rates, with closed circles determined in the mixed-effects model including only homozygous strains and open symbols from the model that includes heterozygous strains (open diamonds: double heterozygotes; open triangles: single heterozygotes that are wild type at the other gene; open circles: single heterozygotes that are homozygous mutants at the other gene). Points and bars are otherwise as in Fig 3 and S6 Fig. All symbols are colored intermediately according to genotype and arrayed along the x-axis so as to lie between the two strains that are genotypically most similar to it. Solid lines indicate significant comparisons in tests run including only homozygous strains, whereas dotted lines are nonsignificant comparisons. See Fig 3 or S6 Fig for further graphical details. Note that the strain erg5/ERG5 erg6/erg6 was later found to be homozygous for the mutation in ERG5, likely due to an LOH event. All underlying raw data and analyses can be found in Dryad [32]. (TIF) [file pbio.1002591.s007.tif]
