# Supplementary material for: Widespread Genetic Incompatibilities between First-Step Mutations during Parallel Adaptation of Saccharomyces cerevisiae to a Common Environment
Source: PLoS Biol. 2017 Jan 23;15(1):e1002591. doi: 10.1371/journal.pbio.1002591 (PMC5256870; doi:10.1371/journal.pbio.1002591)
Supplement: S1 File — (PDF) [file pbio.1002591.s008.pdf]

Supplemental Materials and Methods

Widespread Genetic Incompatibilities Between First-Step Mutations During Parallel Adaptation of *Saccharomyces cerevisiae* to a Common Environment

Jasmine Ono*, Aleeza C. Gerstein, Sarah P. Otto,

*Email: ono@zoology.ubc.ca

Strain construction details

All possible haploid and diploid genotypes were created for each pair of four beneficial mutations (one in each of *ERG3, ERG5, ERG6* and *ERG7*, main text Table 5). Each mutation was initially isolated in the BY4741 haploid background (*MATa his3*Δ*1 leu2*Δ*0 met15*Δ*0 ura3*Δ*0*) and given a Beneficial Mutation Nystatin (BMN) strain number [1]. Each BMN strain was mated to BY4739 (*MAT*α *leu2*Δ*0 lys2*Δ*0 ura3*Δ*0*) (Open Biosystems) to create strains heterozygous for a single ERG mutation, and diploids were positively selected on plates lacking both histidine and lysine. Similarly, diploid non-mutant strains were created by mating BY4741 and BY4739. In each case, single colonies were then grown up on a second selection plate and frozen at -80°C in 15% glycerol.

MATα single mutant strains were isolated by sporulation of the heterozygous diploids. Diploid stock grown on YPD a plate was used to inoculate 10 mL of YPD and grown overnight on a rotor at 30°C. 200 μL of culture was then washed, spread on potassium acetate plates (1% KOAc, 2% agar) and sporulated at 25°C until a sufficient number of tetrads could be observed. The resulting tetrads were dissected by micromanipulation on YPD plates. The spores were allowed to germinate and grown at 30°C for three days before replica plating to test for auxotrophies, mating type, and nystatin growth ability. Auxotrophy was assessed on SC plates lacking the appropriate amino acid. Mating type was tested by replica plating tetrads onto plates containing a lawn of *MATa* or *MAT*α yeast carrying a histidine (*his1-123*) auxotrophy, allowing them to mate, and subsequently testing for mating on a plate lacking arginine, histidine, leucine, lysine, methionine, tryptophan, adenine and uracil (i.e., a plate on which no original haploid strain could grow). Nystatin growth was assessed on YPD + 8 µM nystatin because growth of ancestral strains was not noticeably inhibited on plates with a lower concentration of nystatin. YPD + 8 µM nystatin plates were made by preparing YPD medium with agar as usual, subsequently adding the appropriate amount of 2.7 mM nystatin stock, and mixing by inversion immediately before pouring. All tetrads were verified for 2:2 segregation of auxotrophies and mating type. Once this was confirmed, the spores that showed growth on the nystatin plate and contained the desired *MAT*α *lys2*Δ*0* mutation were frozen at -80°C in 15% glycerol. Throughout strain construction, histidine and lysine auxotrophies were

consistently kept with the same mating types so that all haploid strains were either *MATa his3Δ1* or *MATα lys2Δ0*. The methionine auxotrophy (*met15Δ0*) did not show strong selection on plates lacking methionine and was not tracked.

The haploid *MATα* strains were then mated to the original *MATa* strains to create strains that were either homozygous for one mutation or heterozygous for two mutations. Diploids were selected and frozen as described for the singly heterozygous strains.

The haploid double mutant strains were created through sporulation and dissection of the double heterozygous strains. Three strains (*erg3/ERG3 erg6/ERG6*, *erg3/ERG3 erg7/ERG7*, and *erg6/ERG6 erg7/ERG7*) were struck from frozen on YPD plates and grown at 30°C for 2-3 days. They were sporulated, dissected and checked as described above except that they were moved to 20°C after three days of sporulation. This protocol was repeated for *erg3/ERG3 erg5/ERG5*, except that it was kept at 20°C from the beginning. *erg5/ERG5 erg7/ERG7* would not sporulate under these conditions. To obtain the *MATa* double mutant strain it was sporulated in 10 mL 1% KOAc + amino acids liquid medium at 20°C. In order to obtain the *MATα* double mutant strain, it was sporulated by streaking a patch of cells onto a GNA pre-sporulation plate (5% dextrose, 3% nutrient broth, 1% yeast extract, 2% agar) and growing at 30°C overnight, repeating the streaking and growth on another GNA pre-sporulation plate, and finally sporulating in 2 mL of supplemented sporulation medium (1% potassium acetate, 0.005% zinc sulphate, 2 mg/100 ml uracil, 10 mg/100 ml leucine) on a rotor at 25°C for five days, then moving to 30°C until a sufficient number of tetrads were found. The same procedure was applied to *erg5/ERG5 erg6/ERG6* to obtain the *MATα* double mutant strain. Tetrads were chosen that showed 2:2 segregation of the nystatin resistance (assessed either on YPD + 8 µM nystatin plates, YPD + 10 µM nystatin plates or in a liquid assay), indicative of two double mutant spores and two wildtype spores. Double mutant strains were frozen at -80°C in 15% glycerol. All haploid double mutant strains were confirmed by Sanger sequencing.

We failed to obtain the *MATa erg5 erg6* double mutant strain through crossing and sporulation because the two genes are linked (they are 48 kb apart but flank the centromere of chr XIII). For this strain, transformations were performed using a protocol based on [2]. *MATa erg5* yeast were grown from a single colony in 10 mL YPD at 30°C. The next day, two new 10 mL YPD tubes were inoculated with 500 µL of yeast from the overnight culture and grown at 30°C until reaching an OD_{600} between 0.5 and 0.6. One tube was used for the transformation, and one was used as a negative control. Cells were collected by spinning the cultures down for 5 minutes at 4500 rpm and were washed twice with water using a spin of 10 minutes at 4500 rpm. The yeast were resuspended in 2 mL of cold 1 M sorbitol, spun at 5000 rpm long enough to pellet the cells, the supernatant was removed, and the yeast were resuspended in 1 mL of cold 1 M sorbitol. 80 µL of these cultures were then electroporated, along with either 8 µg of an oligonucleotide designed to contain the *ERG6* SNP of interest (sequence: TTCAAAGAGGCGATTTAGTTCTCGACGTTCGTTGTG-GTGTTGGGGGCCCAGCAAG) or an equal volume of water, using a BioRad Gene Pulser Xcell and the parameters defined in [2]. Immediately after electroporation, 1 mL of YPD was added to the yeast and the cells were incubated for 1 hour at 30°C to recover. The cells were then plated on YPD + 10 µM nystatin plates and incubated at 30°C until

colonies were visible. The insertion of the mutation in *erg6* was verified by Sanger sequencing.

Strains with one heterozygous and one homozygous locus as well as double homozygous strains were created by mating the corresponding single mutant strains or *MATa* double mutant strains to the *MAT*α double mutant strains, as described above.

Segregating mutation in *DSC2*

The original strain with a mutation in *ERG7* also carried a second mutation in the gene *DSC2* (main text Table 5). This mutation was not originally tracked when constructing the strains and it was later identified by Sanger sequencing in all haploid strains constructed from the original strain carrying a mutation in *ERG7* (Table 1). Two combinations of strains that differed in their status at *DSC2* between the mating types (*erg7* and *erg5 erg7*) and were tested for differences in maximum growth. No significant difference was found for growth rate in nystatin whether we treat each replicate as independent or average data points collected on the same day (Welch two sample t-tests with replicates treated as independent, *erg7*: t = -0.38, df = 49.56, *P* = 0.71; *erg5 erg7*: t = -1.01, df = 37.66, *P* = 0.32). In YPD, a significant difference was found only for the gene combination *erg5 erg7* and only when all replicates were treated as independent (Welch two sample t-tests, *erg7*: t = 0.27, df = 50, *P* = 0.79; *erg5 erg7*: t = -2.32, df = 35.97, *P* = 0.026). The test was not significant when data points for each day were averaged (*erg5 erg7*: t = -1.90, df = 7.87, *P* = 0.094). Furthermore, the difference between mutant and wildtype *DSC2* growth rates was in each case minor and did not substantially alter the data points illustrated in main text Fig 3 or the conclusions drawn.

Table 1: ***DSC2* allele status in haploid strains constructed from the original strain carrying a mutation in *ERG7*.**

Strain	Mating Type	Allele status at *DSC2*
erg7	a	mutant
erg7	alpha	wildtype
erg3 erg7	a	mutant
erg3 erg7	alpha	mutant
erg5 erg7	a	mutant
erg5 erg7	alpha	wildtype
erg6 erg7	a	wildtype
erg6 erg7	alpha	wildtype

Preparing stocks for growth rate assays

A total of seven growth rate assays were conducted for our analysis of epistasis. We had originally intended to perform three assays, but four more were performed to maintain the intended level of replication after encountering problems with growth and strain construction. For an overview of which lines were included in which fitness assays, see S1 Table and for complete information about the growth assays, see files deposited at Dryad. Many of the lines involved in this study had poor growth even in a rich medium. Because of this, care was taken to standardize initial cell densities

("pre-assays") for use in subsequent growth rate assays. General methods will be explained first, with exceptions to these methods explained subsequently.

The pre-assay took place in 100-well honeycomb Bioscreen plates using a permissive medium of 148.5 μL of YPD + 0.5 μM nystatin (except for the first assay, which used only YPD). YPD + 0.5 μM nystatin was used to help prevent reversion of strains with severe growth defects in YPD while still permitting the growth of all strains. The wells were inoculated with 1.5 μL of frozen culture. Replicates were randomized within plates, always including all lines on the same plate for a given pair of mutations. The plates were incubated in the Bioscreen C Microbiological Workstation at 30°C with maximum continuous shaking, measuring the optical density (OD) of the cultures every 30 minutes using the wideband filter.The cultures were incubated in this way for 72 hours, which was enough time for most strains to obtain clear growth (defined as a maximum OD of about two times the initial OD); anything below this threshold was excluded from analysis unless otherwise noted. Maximum OD was used to determine the volume to transfer for the growth rate assays. If it was above 1, we transferred 1.5 μL into one plate containing 148.5 μL of YPD and one plate containing 148.5 μL of nystatin2 (using the same randomized well map). If the maximum OD was below 1, it was rounded to the nearest 0.05, and the transferred volume was scaled accordingly (giving final volumes ranging between 150 μL and 156 μL).

To investigate whether the pre-growth medium influenced growth rate, we ran a sign test comparing the mean maximum growth rates in the nystatin2 assay between Assay 1 (in which all strains were pre-grown in YPD) and Assay 2 (in which all strains were pre-grown in 0.5 μM nystatin). All 47 strains that were included in both assays (and not omitted due to growth problems) were included in the sign test, which was run using the function *binom.test* in the package *stats* by counting the number of strains for which maximum growth rate was higher in Assay 2 and comparing that to what is expected by chance (p = 0.5). No significant difference was found ($P = 0.56$; similar results were obtained with a paired t-test: $P = 0.24$).

The *erg6/erg6 erg7/erg7* diploid strain showed consistently poor growth, and all of the data for this strain comes from the fourth and sixth assays where the pre-assay was conducted over a longer period of time in a larger volume of liquid in an attempt to initiate the assays with the same number of cells. Briefly, 10 mL of 0.5 μM nystatin in a test tube was inoculated with 15 μL of *erg6/erg6 erg7/erg7* from frozen two days before all other lines were inoculated from frozen. The tube was incubated at 30°C on a rotor for this time. On the day when all other strains were being inoculated from frozen, the 10 mL tube of *erg6/erg6 erg7/erg7* was spun down in multiple 1.5 mL tubes and concentrated into 500 μL in one tube. 150 μL of this concentrated culture was used to fill the appropriate wells of the pre-assay plate. Despite this extra growth time and concentrating of cells, *erg6/erg6 erg7/erg7* still did not grow to an OD above the threshold at the end of the pre-assay in one of the two cases where growth rate was measured for this line and only barely did so in the other. Yeast was added to the assay plates from these wells according to their measured OD after the growth phase even though the OD was below the threshold (up to 7.5 μL was transferred).

We also modified growth conditions for three other strains that showed poor growth in early pre-assays (*MATa erg6 erg7*, *MATα erg6 erg7*, and *erg6/erg6*). Once low growth from frozen was established, 2 μL (rather than 1.5 μL) of frozen stock was used to inoculate the wells in the pre-assay plates. Backup tubes were also grown for these strains and described when used. In all cases, backup tubes that contained YPD + 0.5 μM nystatin as the growth medium were inoculated from frozen at the same time as the pre-assay plates and were incubated at 30 °C, shaking at 200 rpm.

In the pre-assay for the second growth rate assay, two out of four replicates of *MATα erg6 erg7* had still not grown to an OD above the threshold by 72 hours. One well was omitted. For the other well, 1 mL from a 10 mL backup tube (originally inoculated with 10 μL of frozen culture) was spun down at 3000 rpm for 3 minutes, and this concentrated culture was used to replace the 150 μL on the growth plate. New OD readings were taken, and the new OD was within the range measured for the other strains.

In the third pre-assay, four out of four *MATα erg6 erg7* wells were below the threshold for detecting growth after 72 hours. The liquid from the wells was replaced with culture from four 10 mL backup tubes (originally inoculated with 10 μL of frozen culture). After measuring the OD of these wells, one well was still not above the threshold; to ensure that enough cells were transferred for that one line, we concentrated the cells found in 1 mL of the culture from the corresponding tube by spinning them down using a tabletop centrifuge and removing most of the supernatant, leaving ~200 μL of concentrated culture. 1.5 μL of this culture was transferred directly to the honeycomb plate for the growth assay.

In the sixth pre-assay, one replicate of *MATα erg6 erg7* remained below the threshold for growth after 72 hours. The liquid from the well was replaced with culture from a 1.5 mL backup tube containing 500 μL of culture (originally inoculated with 5 μL of frozen culture). A new OD reading was taken of that well and was within the range measured for the other strains.

Following each pre-assay, growth rate assays were conducted in both YPD and YPD + 2 μM nystatin ('nystatin2'), as described in the main text.

Analysis including outliers

All qualitative relationships between strains and the main conclusions were insensitive to the exclusion or inclusion of the identified outliers, with two exceptions for the haploids in nystatin2 (see S6 Fig and S7 Fig for versions of Fig 3 and Fig 4 that include all outliers). One exception is that the *erg3 erg5* strain no longer had a significantly lower maximum growth rate than the *erg3* strain in nystatin2. This was due to one large outlier in the *MATa erg3* data, which exhibited almost no growth (maximum growth rate of 0.038), while all remaining wells (including both mating types) showed substantial growth (maximal growth rate ranged from 0.16 to 0.25 across 35 wells). The exclusion of this single outlier leads to the observation of a significant difference between the aforementioned strains.

A similar failure of one well to show substantial growth was observed in *erg6/erg6* and *erg3/erg3 erg6/ERG6*. In addition, two wells of *erg6/ERG6* showed substantially higher growth (0.17 and 0.19), compared to all remaining wells (0.0022 to 0.072 across 22 wells), although our outlier exclusion algorithm only allowed one point to be excluded per line. These other examples did not affect the statistical results but suggest either occasional contamination or mutation.

The other statistical difference is that the *erg3 erg6* strain no longer had a significantly lower maximum growth rate than the *erg6* strain in nystatin2. The difference between these strains is only slightly significant in the model excluding all outliers ($P = 0.047$) and becomes marginal when either including all outliers ($P = 0.083$) or excluding only the one outlier replicate of *erg3* ($P = 0.058$). We believe that this represents a lack of power to detect a true, small difference in the haploids as this relationship is supported in the homozygous diploids (excluding outliers: $P = 0.0069$; including outliers: $P = 0.041$). For the full alternative analysis without outlier removal, see Dryad file.

References

[1] Gerstein AC, Lo DS, Otto SP. Parallel genetic changes and nonparallel gene-environment interactions characterize the evolution of drug resistance in yeast. Genetics. 2012;192(1):241–252.

[2] Cregg JM. DNA-mediated transformation. *Pichia* Protocols. 2007; p. 27–42.
