# Supplementary material for: Widespread Genetic Incompatibilities between First-Step Mutations during Parallel Adaptation of Saccharomyces cerevisiae to a Common Environment
Source: PLoS Biol. 2017 Jan 23;15(1):e1002591. doi: 10.1371/journal.pbio.1002591 (PMC5256870; doi:10.1371/journal.pbio.1002591)
Supplement: S1 Table — In each epistasis assay, growth rate was measured in a Bioscreen C over a 24-hour period for a pair of ergosterol mutations (first column) using two replicate wells for each genotype (ancestral, single mutant, double mutant for haploids, including those heterozygous or homozygous for diploids), with the exception of double mutant haploids, which were measured in four replicate wells. Checkmarks indicate that all data from this assay were used, whereas bullets indicate that some strains were omitted (see footnotes). (PDF) [file pbio.1002591.s009.pdf]

| Mutation Pair | Assay 1 | Assay 2[a] | Assay 3 | Assay 4 | Assay 5 | Assay 6 | Assay 7 |
|---|---|---|---|---|---|---|---|
| erg3 & erg5 | ✓ | ✓ | ✓ | | | | |
| erg3 & erg6 | ✓ | ✓ | ✓ | | | | |
| erg3 & erg7 | ✓ | ✓ | ✓ | | | | |
| erg5 & erg6 | *MAT*α only[b] | *MAT*α only[b] | *MAT*α only[b] | •[c] | •[c] | •[c] | ✓[d] |
| erg5 & erg7 | •[e] | •[e] | •[e] | ✓ | [f] | ✓ | |
| erg6 & erg7 | •[g] | •[g] | •[g] | ✓ | [h] | ✓ | |

[a] The Bioscreen bulb burned out during the pre-assay; OD readings taken after replacing the bulb were used to estimate the final cell densities for inoculation of the growth rate assays.

[b] Others not assayed because *MATa erg5 erg6* was unavailable.

[c] *erg5/erg5 erg6/erg6* data from this assay were omitted from final analyses (see [d]).

[d] Because the double homozygous mutant displayed high levels of growth in both YPD and nystatin, we were concerned that the stock might contain a mixture of resistant and non-resistant cells. We thus struck this stock down to colonies on a YPD plate, picked five colonies, and used the resulting five stocks to assay growth. Each stock was confirmed to be *erg5/erg5 erg6/erg6* by Sanger sequencing. For each of these stocks, two replicate wells (1 stock) or three wells (4 stocks) were used to measure growth. All mutant and non-mutant combinations of *erg5* and *erg6* (haploid and diploid) were also regrown in two replicate wells in this assay. The growth rates of the diploid double mutants did not differ qualitatively from previous assays in nystatin but were substantially lower in YPD, consistent with the population analyzed in previous assays having been polymorphic (allowing non-resistant cells to proliferate). Thus, only data from Assay 7 was used for the growth rate of *erg5/erg5 erg6/erg6*, although the qualitative results in nystatin are unaffected if all data were used. Data from each well were treated independently in the analysis, given that each was grown separately from frozen.

[e] *erg5/erg5 erg7/ERG7* data from this assay were omitted from final analyses due to an error in the creation of the original line (identified by Sanger sequencing).

[f] Data not collected due to Bioscreen machine error resulting in lower replication for the line *erg5/erg5 erg7/ERG7*.

[g] *erg6/erg6 erg7/erg7* and some *erg6 erg7* haploid data could not be used due to insufficient starting cell densities of the double mutant lines from the pre-assays so that appreciable growth was never observed.

[h] Data not collected due to machine error. Epistasis was large and easy to detect for this gene pair, despite the lower replication.
